# Mitochondrial DNA damage as a potential biomarker of LRRK2 kinase activity in LRRK2 Parkinson’s disease

**DOI:** 10.1038/s41598-020-74195-6

**Published:** 2020-10-14

**Authors:** C. P. Gonzalez-Hunt, E. A. Thacker, C. M. Toste, S. Boularand, S. Deprets, L. Dubois, L. H. Sanders

**Affiliations:** 1 Department of Neurology, Duke University Medical Center, Durham, NC 27710 USA; 2 Rare & Neurologic Diseases Research, Sanofi, Chilly Mazarin, France

**Keywords:** Neurological disorders, Neurodegenerative diseases, Parkinson's disease

## Abstract

Leucine-rich repeat kinase 2 (LRRK2) is a promising therapeutic target for the treatment of Parkinson’s disease (PD) and LRRK2 kinase inhibitors are currently being tested in early phase clinical trials. In order to ensure the highest chance of success, a biomarker-guided entry into clinical trials is key. LRRK2 phosphorylation, and phosphorylation of the LRRK2 substrate Rab10, have been proposed as target engagement biomarkers for LRRK2 kinase inhibition. However, a pharmacodynamic biomarker to demonstrate that a biological response has occurred is lacking. We previously discovered that the LRRK2 G2019S mutation causes mitochondrial DNA (mtDNA) damage and is LRRK2 kinase activity-dependent. Here, we have explored the possibility that measurement of mtDNA damage is a “surrogate” for LRRK2 kinase activity and consequently of kinase inhibitor activity. Mitochondrial DNA damage was robustly increased in PD patient-derived immune cells with LRRK2 G2019S mutations as compared with controls. Following treatment with multiple classes of LRRK2 kinase inhibitors, a full reversal of mtDNA damage to healthy control levels was observed and correlated with measures of LRRK2 dephosphorylation. Taken together, assessment of mtDNA damage levels may be a sensitive measure of altered kinase activity and provide an extended profile of LRRK2 kinase modulation in clinical studies.

## Introduction

Mutations in leucine-rich repeat kinase 2 (*LRRK2*) are the most common genetic cause of autosomal-dominant Parkinson’s disease (PD), accounting for about 3–4% of all PD^1,2^. *LRRK2* G2019S is the most frequent pathogenic missense mutation, which is thought to confer a toxic gain-of-function in LRRK2 kinase activity and has been strongly implicated in PD pathogenesis and neuronal cell death^3–6^. Increased LRRK2 kinase activity appears to be a shared feature of all the known pathogenic missense LRRK2 mutations^7^. Consistent with these findings, a neuroprotective effect of LRRK2 inhibitors has been demonstrated in PD-relevant cell and rodent models^8^. In addition to inherited *LRRK2* mutations, the *LRRK2* locus contains a risk factor for idiopathic PD^9^. Wild-type LRRK2 may play an important role in at least a subset of idiopathic PD, as we recently showed that LRRK2 was abnormally activated in substantia nigra dopamine neurons in post-mortem brain tissue from subjects with idiopathic PD^10^. Given that LRRK2-associated PD patients also present with clinical and neuropathological profiles largely indistinguishable from late-onset idiopathic PD, an exciting prospect is that LRRK2 kinase inhibitors may have broader applicability to the idiopathic PD population^3^.

There have been focused efforts to develop LRRK2 kinase activity inhibitors with small-molecule compounds^11–15^. Multiple LRRK2 kinase inhibitor compounds are currently being tested in clinical trials. Despite the great progress of LRRK2 kinase inhibitors in late stages of preclinical evaluation and more recently in early phase clinical trials, there are emerging suitable biomarkers but not yet validated readouts for target engagement, efficacy, or LRRK2-mediated pathobiology. In order to achieve neuroprotection in clinical trials, it is essential to provide a biomarker-guided entry of LRRK2 kinase inhibitors in PD patients^16^.

Currently, the most widely used tool for measuring LRRK2 kinase inhibition is the phosphorylation levels of LRRK2 on residue Ser935^17–19^. This is accomplished by measuring protein levels in biofluids such as blood-derived cells, urine, and cerebrospinal fluid (CSF) via immunoassays, though the detection of endogenous levels of this epitope can be challenging given the low levels of expression^16,20^. Levels of LRRK2 Ser935 cannot distinguish between healthy controls and PD patients basally or following LRRK2 kinase inhibitor treatment, limiting its use as a patient stratification biomarker^17,18^. Although phosphorylation of LRRK2 Ser935 is required for the binding of LRRK2 to 14-3-3 family adaptor proteins, LRRK2 Ser935 regulation or the 14-3-3 interaction effects on LRRK2 function are unclear^21^. Paradoxically, mutations that ablate or increase LRRK2 kinase activity do not alter the abundance of this constitutive phosphorylation^22–26^. While useful, LRRK2 Ser935 is an indirect readout and does not always reflect LRRK2 protein kinase activity and therefore caution should be taken if used as a single measure of target engagement in clinical trials. Due to this complexity, additional distinct biomarkers including the *bona fide* LRRK2 substrates (*e.g.* LRRK2 Ser1292 and Rabs) have been pursued.

The autophosphorylation of LRRK2 at Ser1292 has been proposed to be a direct indicator of kinase activity^14^. Due to low levels, however, measuring endogenous LRRK2 Ser1292 has been technically challenging and only robustly detected in overexpression models or in urine following exosome enrichment in PD patients carrying the G2019S mutation or idiopathic PD^27,28^. Recently, a new fractionation-based enrichment technique has made measuring endogenous LRRK2 Ser1292 via immunoblotting successful in G2019S but not wild-type tissue, and validating this technique in patient-derived material will help determine the applicability in the clinic ^29^. LRRK2 directly phosphorylates a subset of Rab GTPase family members, and to date, the most robust Rab substrate of LRRK2 is Rab10^30^. Rab10 phosphorylation is decreased in subjects with PD and healthy controls in response to LRRK2 kinase inhibition, showing promise as a biomarker of target engagement^18,31,32^. However, Rab10 phosphorylation does not correlate with LRRK2 levels or distinguish between PD patients and controls, limiting its utility as a patient enrichment biomarker^32,33^. This suggests a potential dissociation between LRRK2 activity and levels and further elucidation is needed to better understand the conditions in which LRRK2 phosphorylates Rab10 and how this may impact its use as a biomarker. With drugs targeting LRRK2 already in clinical trials, it is of utmost importance that robust and sensitive biomarkers are developed, in particular, pharmacodynamic biomarkers for LRRK2 kinase inhibition.

LRRK2 kinase activity mediates PD-associated pathogenic phenotypes, including mitochondrial dysfunction^16^. We recently showed that mitochondrial DNA (mtDNA) damage is increased in LRRK2 G2019S mutation carriers and can be abrogated either by gene correction of the G2019S mutation or with LRRK2 kinase inhibition^34,35^. Based on these aforementioned findings, we considered that mtDNA damage levels may be useful for measuring the biological response or effectiveness of LRRK2 kinase inhibitors. In this study, we investigated the correlation between reversal of LRRK2 G2019S-induced mtDNA damage and inhibition of LRRK2 kinase activity. The time course and concentration-dependence of two novel and one well-established LRRK2 kinase inhibitor and their effect on the mtDNA phenotype in healthy control and PD LRRK2 G2019S patient-derived cells were examined. Measuring the reversal of mtDNA damage levels is an innovative and readily available tool to measure LRRK2 kinase inhibition as a potential pharmacodynamic biomarker in clinical studies, and might have broader applications that extend to other PD-modifying drugs.

## Results

### LRRK2 inhibitors are not toxic to human lymphoblastoid cells

**Figure 1:**
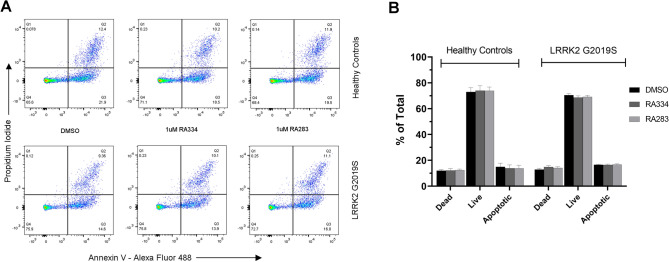
Novel LRRK2 kinase inhibitors are not acutely toxic to human lymphoblastoid cells. Human lymphoblastoid cells derived from healthy control or LRRK2 G2019S carriers were treated with the highest dose used in the overall study (1 μM) of the LRRK2 kinase inhibitors RA334 or RA283 and compared to DMSO treated cells. Apoptosis was assessed by Annexin-PI staining and flow cytometry. (**A**) Representative flow cytometry distribution of live, apoptotic or dead cells. (**B**) Quantification of Annexin-PI staining reveals LRRK2 kinase inhibitors do not cause toxicity relative to vehicle treated cells. Data are mean ± SEM. n = 2 biological replicates (4 cell lines total) performed at least in technical triplicate.

Cell pellets from the peripheral blood mononuclear cell (PBMC) fraction derived from human blood, which contains mainly lymphocytes, are routinely obtained and considered for target engagement and other purposes for LRRK2-targeting therapies in the clinic. Therefore, to evaluate mtDNA damage for use as a pharmacodynamic biomarker of LRRK2 kinase inhibitors in clinical trials and compare it to other candidate target engagement biomarkers, we examined healthy control and PD LRRK2 G2019S patient-derived Epstein-Barr virus (EBV)-transformed lymphoblastoid cell lines (LCL); detailed demographic information can be found in Supplemental Table S1. We first assessed the induction of apoptosis with two novel LRRK2 kinase inhibitors in human healthy control and LRRK2 G2019S patient-derived LCLs (For data comparing RA334 and RA283 to LRRK2 kinase inhibitor tool compounds, please see Supplemental Table S2). Cells were incubated with LRRK2 kinase inhibitors for the longest exposure time (24 h) and with the maximum dose used in this study (1 µM). Neither of the two distinct LRRK2 inhibitors (RA334 or RA283) had an acute toxic effect on cell viability. The percentage of apoptotic cells was similar in LRRK2 G2019S patient-derived LCLs compared to healthy controls (Fig. 1). The apoptosis rates did not change after treatment with the LRRK2 kinase inhibitors RA334 or RA283 in either the healthy control or LRRK2 G2019S patient-derived LCLs (Fig. 1). Consistent with previously published studies^36^, these results suggest that LRRK2 kinase inhibitors do not cause acute toxicity in human LCLs, regardless of disease status.

### A similar decrease in LRRK2 phosphorylation in control and LRRK2 G2019S patient-derived cells with LRRK2 inhibitor exposure

**Figure 2:**
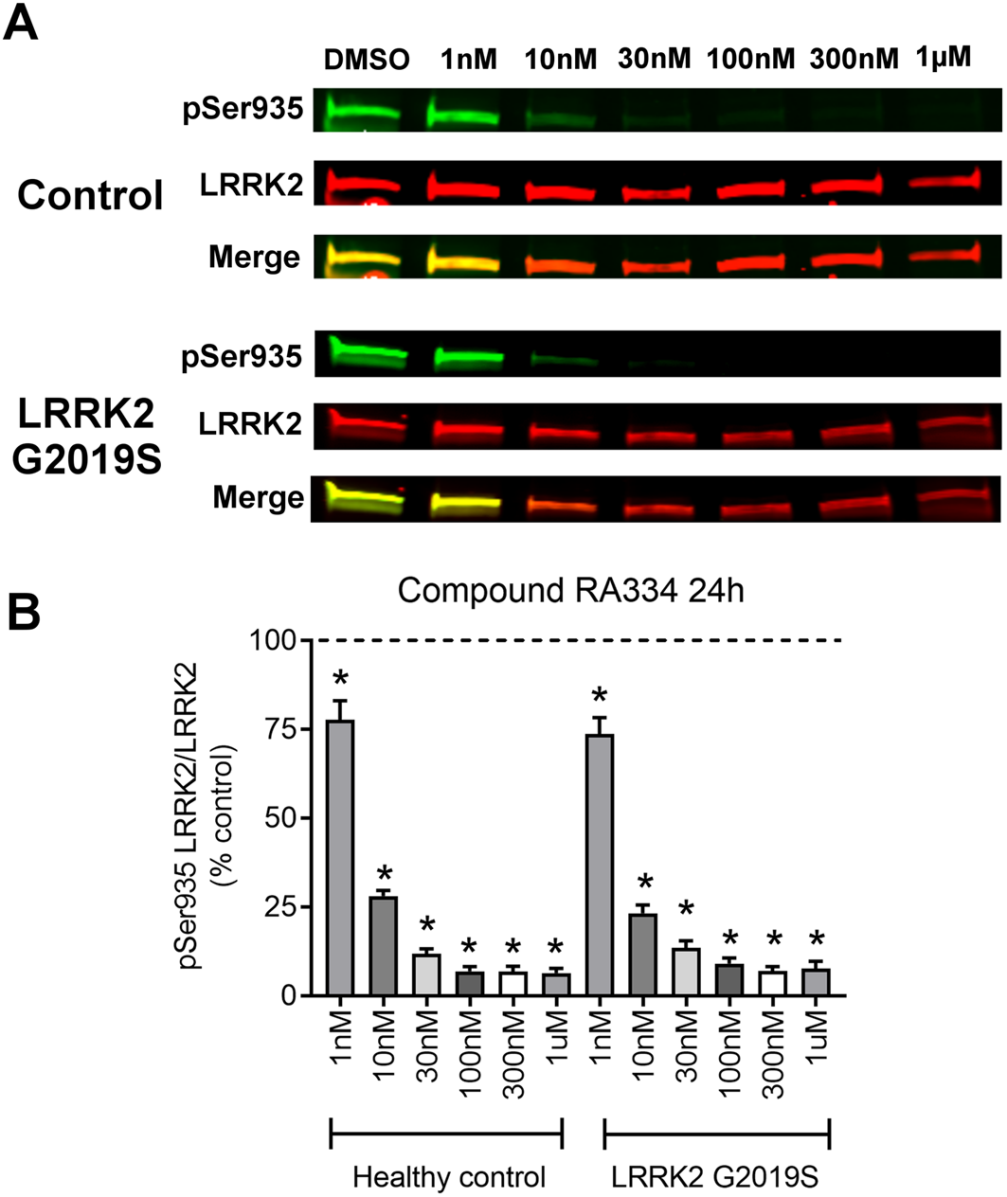
Dose–response curve of RA334 on LRRK2 Ser935 dephosphorylation in control and LRRK2 G2019S PD patient LCLs. (**A**) Representative western blots of healthy control and LRRK2 G2019S patient-derived LCLs treated for 24 h with Compound RA334. (**B**) Quantification of western blots demonstrated that Compound RA334 decreased LRRK2 pSer935 levels at all doses tested. Data are mean ± SEM. (*p < 0.001, determined by one-way ANOVA with a Tukey’s post-hoc comparison). n = 3 biological replicates (6 cell lines total), each performed in technical replicate. Full blots available in Supplemental Figs. S7 and S8.

**Figure 3:**
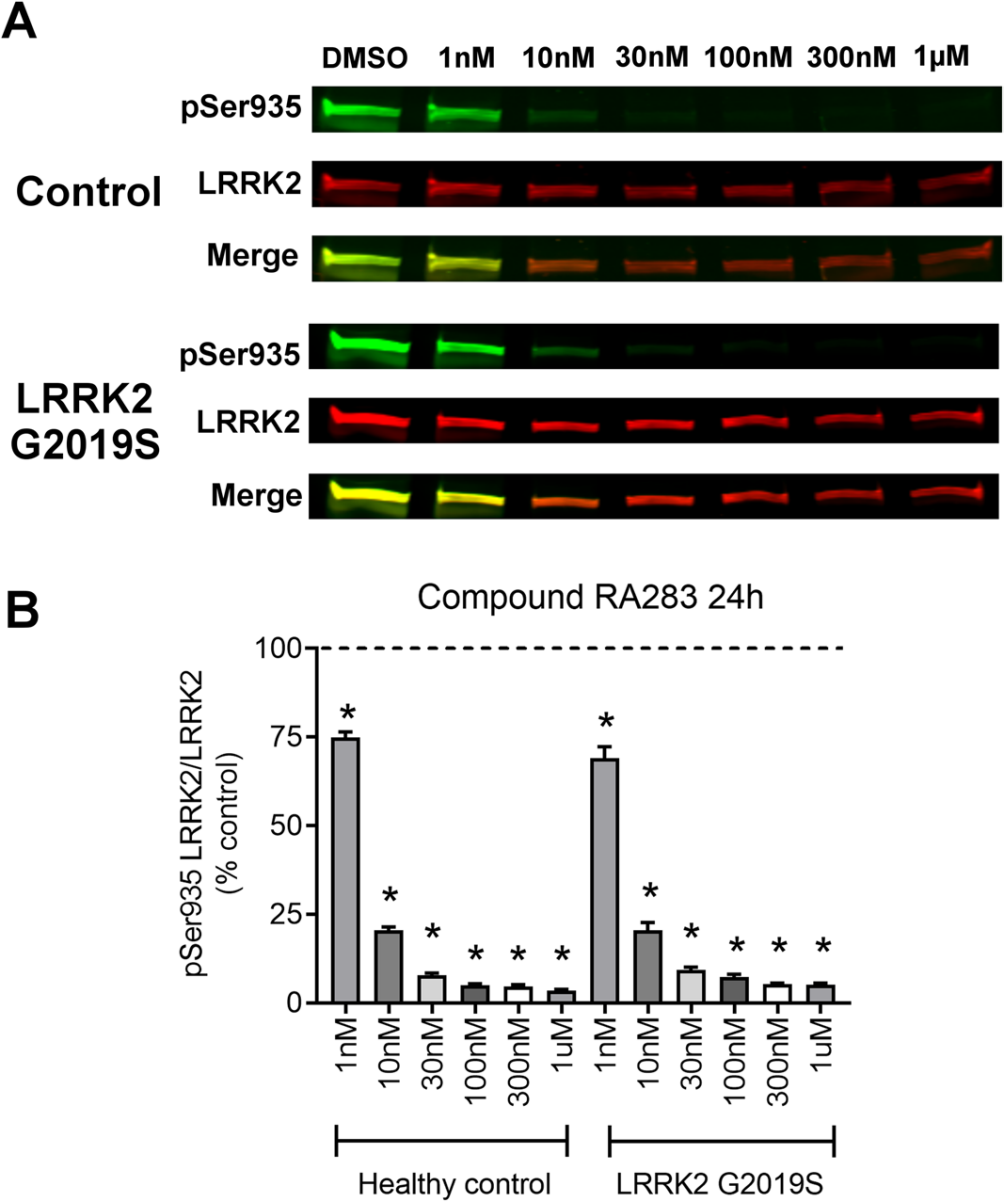
Dose–response curve of RA283 on LRRK2 Ser935 dephosphorylation in control and LRRK2 G2019S PD patient LCLs. (**A**) Representative western blots of healthy control and LRRK2 G2019S patient-derived LCLs treated for 24 h treatment with Compound RA283. (**B**) Quantification of western blots demonstrated that Compound RA283 decreased LRRK2 pSer935 levels at all doses tested. Data are mean ± SEM. (*p < 0.001, determined by one-way ANOVA with a Tukey’s post-hoc comparison). n = 3 biological replicates (6 cell lines total), each performed in technical replicate. Full blots available in Supplemental Figs. S9 and S10.

We next assessed whether LRRK2 inhibitors, RA334 or RA283, reduced phosphorylation of LRRK2 Ser935 to a similar extent in healthy controls and LRRK2 G2019S patient-derived LCLs. To do this, a dose–response experiment was conducted with compounds RA334 or RA283 at concentrations ranging from 1 nM–1 μM. At the lowest concentration tested for compound RA334 (1 nM), LRRK2 Ser935 phosphorylation was decreased by approximately 25% (Fig. 2). Exposure to RA334 at 100 nM and above, almost completely ablated LRRK2 Ser935 phosphorylation (Fig. 2). The dose–response curve of reduction in LRRK2 Ser935 phosphorylation with compound RA283 was similar to the results with compound RA334. LRRK2 Ser935 was decreased by approximately 25% following treatment with 1 nM of RA283. Maximal dephosphorylation of LRRK2 Ser935 was also achieved at 100 nM of compound RA283 (Fig. 3).

**Figure 4:**
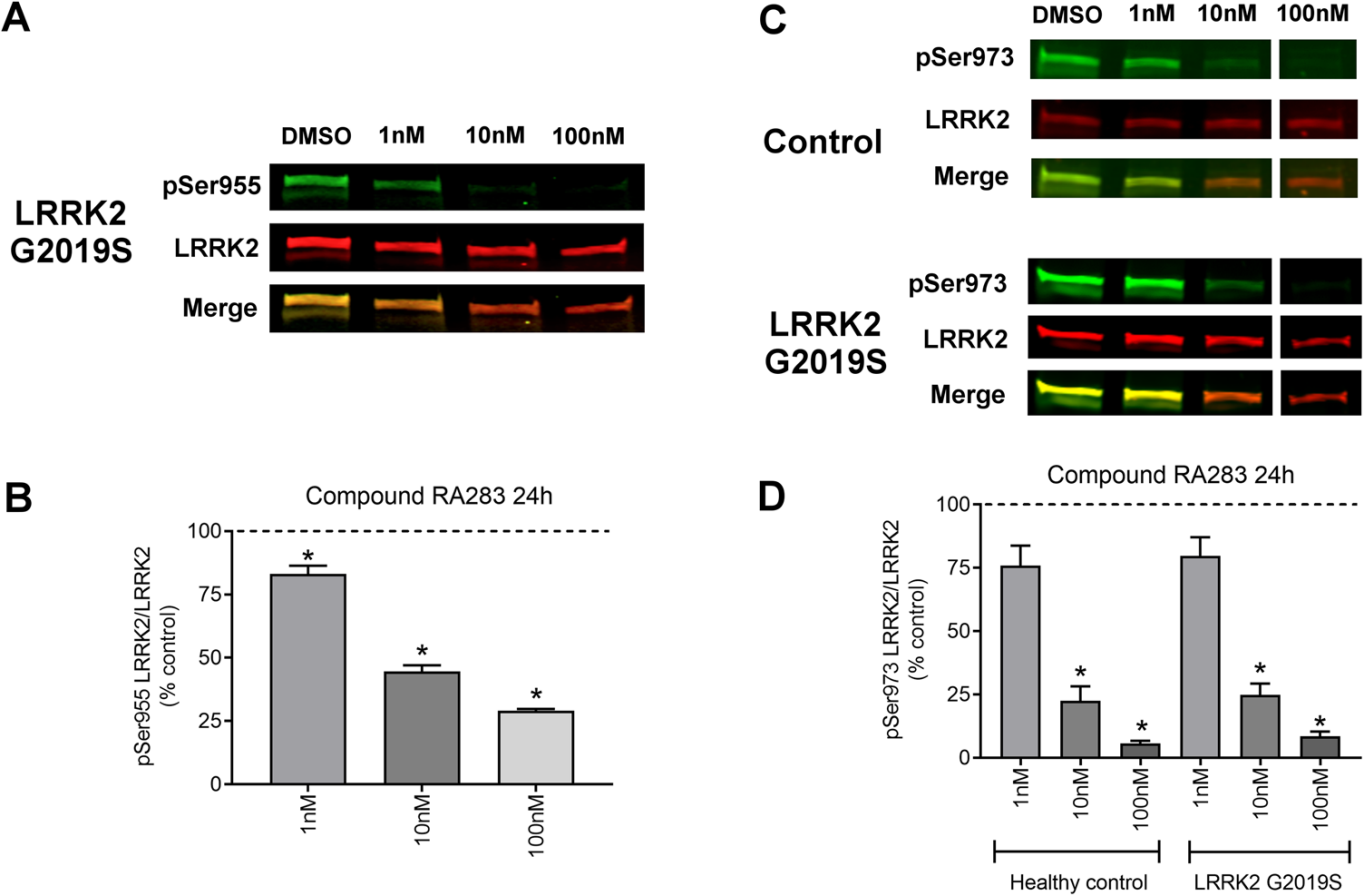
The effect of LRRK2 kinase inhibition on constitutive LRRK2 Ser955 and Ser973 phosphorylation sites. (**A**) Representative western blot of LRRK2 G2019S patient-derived LCLs treated for 24 h with Compound RA283. (**B**) Quantification of western blots demonstrated that Compound RA283 decreased LRRK2 pSer955 levels at all doses tested. (**C**) Representative western blots of LRRK2 G2019S patient-derived LCLs treated for 24 h with Compound RA283. White space represents where sections of the blot were removed for clarity. (D) Quantification of western blots demonstrated that Compound RA283 decreased LRRK2 pSer973 levels at all doses tested. Data are mean ± SEM. (*p < 0.001, determined by one-way ANOVA with a Tukey’s post-hoc comparison). n = 3 (ND00011 and ND00264) technical replicates for panels A and B; n = 2 (ND00011 and ND00264) plus n = 1 (ND02559 and ND01618) technical replicates for (**C**,**D**). Full blots available in Supplemental Figs. S11, S12, and 13.

To determine if LRRK2 kinase inhibitors similarly reduced LRRK2 phosphorylation at other constitutive sites (specifically Ser955 and Ser973), healthy control and LRRK2 G2019S patient-derived LCLs were treated with compound RA283. Based on the previous dose–response experiments, the LRRK2 kinase inhibitor RA283 was used at concentrations of 1, 10, and 100 nM. Reduction in the phosphorylation of LRRK2 Ser955 occurred at all doses tested in LRRK2 G2019S patient-derived LCLs, but the decrease was less than that observed for LRRK2 Ser935 at the same doses (Fig. 4A,B). Levels of LRRK2 pSer955 in healthy control LCLs were below detection and did not permit quantification, potentially requiring an additional immunoprecipitation step as previously reported^37,38^ (Supplemental Fig. S1). While the maximal reduction of phosphorylation of LRRK2 Ser973 was found at 100 nM, there was a lack of a detectable significant decrease in LRRK2 Ser973 phosphorylation at the lowest dose tested (1 nM) with compound RA283 exposure (Fig. 4C,D). Overall, the least variance was obtained with the LRRK2 Ser935 site, with the most variance observed with the LRRK2 Ser973 site. The LRRK2 Ser935 residue was also a more sensitive readout of LRRK2 kinase inhibitor activity, relative to LRRK2 Ser955 or LRRK2 Ser973. Therefore, LRRK2 Ser935 was assayed for the remainder of the experiments.

### Duration of LRRK2 kinase inhibitor exposure does not change LRRK2 Ser935 dephosphorylation kinetics

**Figure 5:**
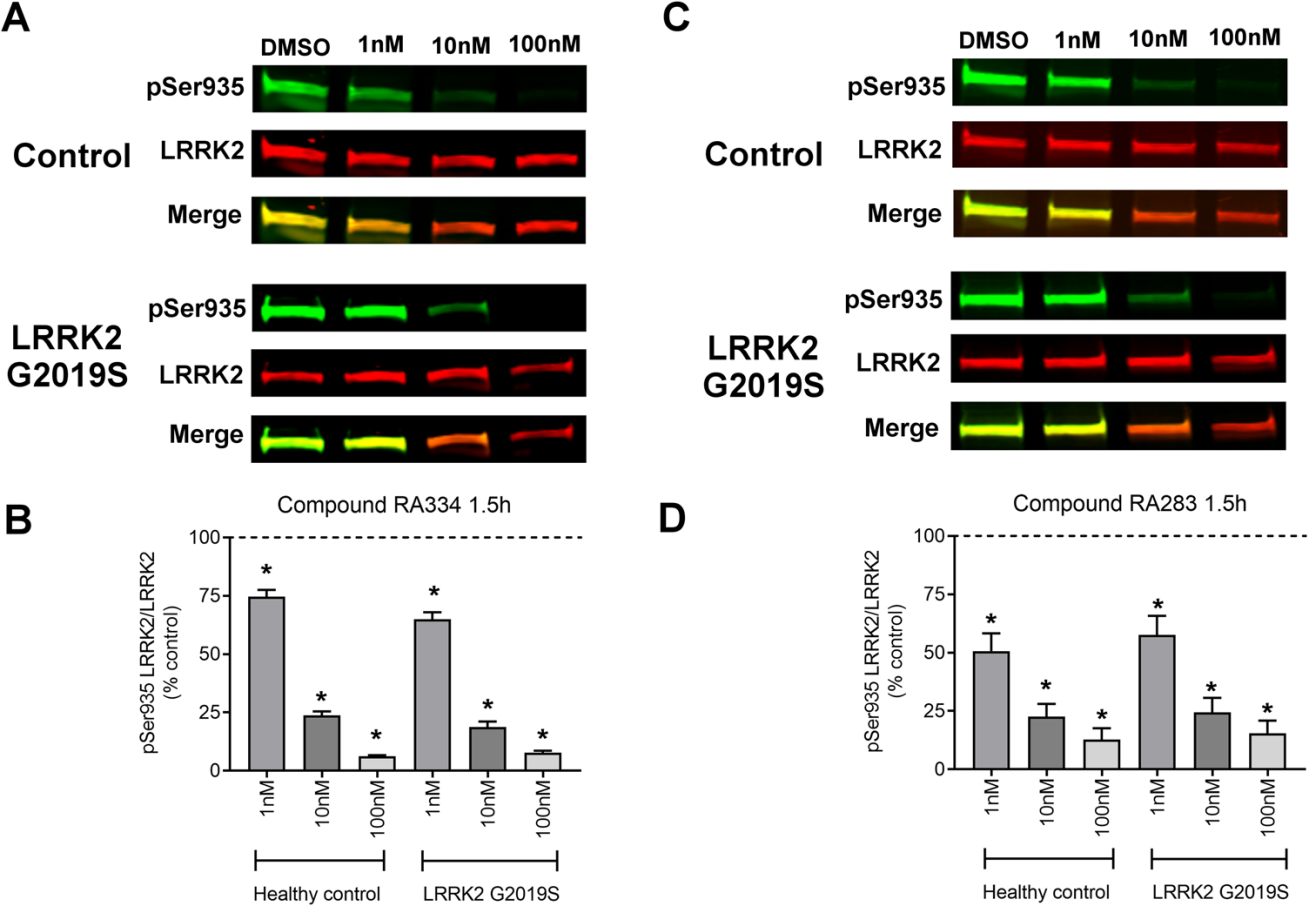
Acute LRRK2 kinase inhibition. (**A**) Representative western blots of healthy control and LRRK2 G2019S patient-derived LCLs treated for 1.5 h with RA334. (**B**) Quantification of western blots demonstrated that RA334 decreased LRRK2 pSer935 levels at all doses tested. (**C**) Representative western blots of healthy control and LRRK2 G2019S patient-derived LCLs treated for 1.5 h with RA283. (**D**) Quantification of western blots demonstrate that RA283 decreased LRRK2 pSer935 levels at all doses tested. All experiments were performed with at least three biological replicates (6 cell lines total), each performed in technical replicate. Data are presented as mean ± SEM. (*p < 0.001, determined by one-way ANOVA with a Tukey’s post-hoc comparison). Full blots available in Supplemental Figs. S14 and S15.

We next determined whether an acute or shorter exposure to LRRK2 kinase inhibitors affected LRRK2 pSer935 reduction similarly in healthy controls and LRRK2 G2019S patient-derived LCLs. Based on the optimized dose–response experiments performed at 24 h (see Figs. 2 and 3), controls and LRRK2 G2019S patient-derived LCLs were treated with 1, 10, and 100 nM of either compound RA334 or RA283 for 1.5 h. At the lowest concentration tested for compound RA334 (1 nM), LRRK2 pSer935 was decreased by approximately 25% (Fig. 5A,B). Exposure to RA334 at 10 nM reduced LRRK2 Ser935 phosphorylation approximately by 75% (Fig. 5A,B). The dose–response curve of reduction in LRRK2 Ser935 phosphorylation with compound RA283 was similar to the results with compound RA334, except that LRRK2 pSer935 was decreased about 50% following treatment with 1 nM of RA283 (Fig. 5C,D). Overall the response to LRRK2 kinase inhibition by reduction of LRRK2 pSer935 was similar between a short (1.5 h) and longer (24 h) exposure with compounds RA334 and RA283. To compare to an established LRRK2 kinase inhibitor, healthy controls and LRRK2 G2019S patient-derived LCLs were treated with 1, 10, and 100 nM of compound MLi-2 or vehicle for 1.5 h^39^. At the lowest concentration tested for compound MLi-2 (1 nM), LRRK2 pSer935 was decreased by approximately 45% similarly between healthy controls and LRRK2 G2019S patient-derived LCLs (Supplemental Fig. S2). Exposure to MLi-2 at 100 nM reduced LRRK2 Ser935 phosphorylation almost completely (Supplemental Fig. S2). Taken together, the dose–response curve of reduction in LRRK2 Ser935 phosphorylation was similar with RA283, RA334 and MLi-2 and did not differ between PD LRRK2 G2019S patient-derived cells and control cells.

### Rab8 and Rab10 phosphorylation in control and LRRK2 G2019S patient-derived cells

A subset of the Rab GTPase family members, in particular Rab10 and Rab8, have been identified as direct substrates of LRRK2^40^. Therefore, we investigated whether pRab10 or pRab8 level was elevated with the LRRK2 G2019S mutation in patient-derived cells. Consistent with previous reports^18,33^, there was no significant difference in levels of pRab10 between PD LRRK2 G2019S patient-derived and healthy control cells (Supplemental Fig. S3). Similarly, levels of pRab8 were not different in PD LRRK2 G2019S patient-derived and healthy control cells (Supplemental Fig. S4).

### LRRK2 kinase inhibitors moderately reduce LRRK2 expression

Previous studies have shown that LRRK2 kinase inhibition can result in loss of LRRK2 protein due to proteasomal degradation or other unidentified mechanisms^41^. Therefore, we investigated whether compound RA283 affected total levels of LRRK2 in human healthy control-derived LCLs after a 24 h exposure. Levels of total LRRK2 were unchanged in cells treated with 1 nM RA283 as compared to vehicle-treated cells, as shown in Supplemental Fig. S5. In cells treated with 10 or 100 nM of RA283, levels of total LRRK2 were significantly decreased by approximately 25% as shown in Supplemental Fig. S5.

### Reversal of PD LRRK2 G2019S-induced mtDNA damage occurs with novel LRRK2 kinase inhibitors

**Figure 6:**
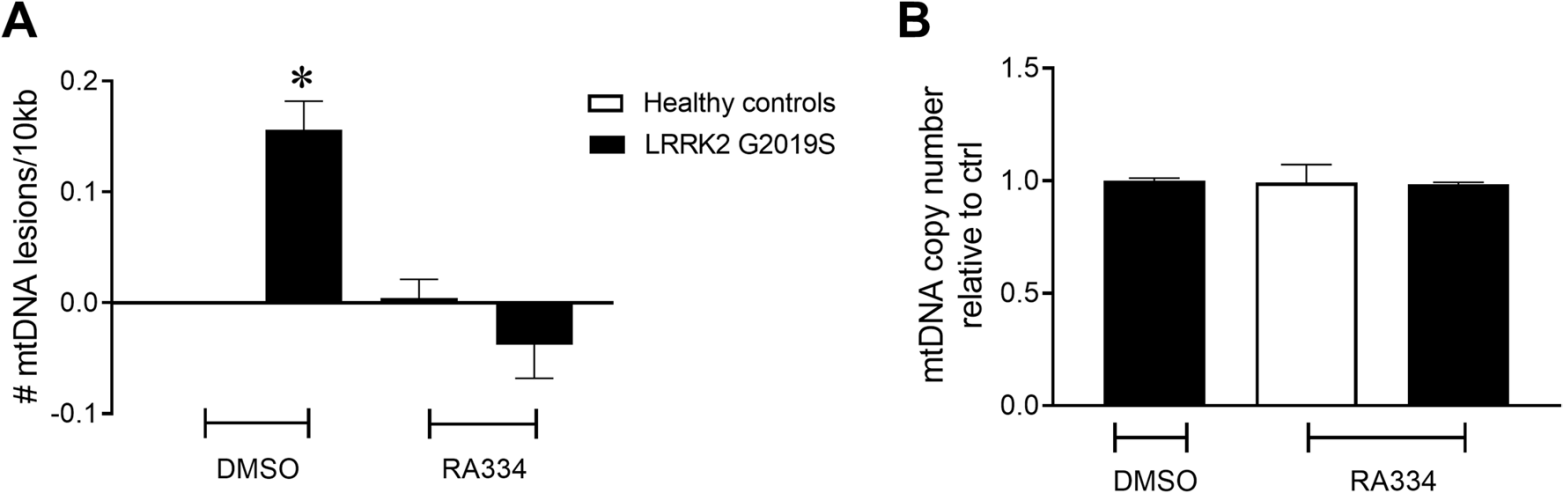
LRRK2 kinase inhibitor exposure restored mtDNA damage to basal levels. (**A**) 24 h treatment with RA334 (1 μM) reversed mtDNA damage in LRRK2 G2019S-patient derived LCLs. (**B**) mtDNA copy number does not change with LRRK2 G2019S mutation or treatment. The PCR-based assay was performed in technical triplicate for each biological replicate. (*p < 0.001, determined by one-way ANOVA with a Tukey’s post-hoc comparison). n = 3 biological replicates (6 cell lines total), each performed in technical replicate. Data are presented as mean ± SEM.

We recently found that mtDNA damage was increased in LRRK2 G2019S patient-derived LCLs compared to age-matched healthy controls^34^. Exposure of LRRK2 G2019S patient-derived LCLs to a high concentration of the LRRK2 kinase inhibitor, GNE-7915, restored mtDNA damage to control levels with 24 h of exposure^34^. Based on these findings, we first tested whether treating cells with RA334, a distinct LRRK2 kinase inhibitor from GNE-7915, similarly reversed mtDNA damage. Using our PCR-based DNA damage assay, DNA from cell pellets treated with a high concentration of RA334 (1 µM) was evaluated for mtDNA damage. Consistent with our previous findings^34^, baseline mtDNA damage was increased in LRRK2 G2019S patient-derived LCLs relative to healthy controls (Fig. 6A). Of note, similar to LRRK2 G2019S patient-derived LCLs^34^, variability was low among control lines (Supplemental Fig. S6). Culturing in the presence of RA334 restored mtDNA damage to control levels with 24 h of exposure (Fig. 6A). Mitochondrial DNA copy number was not different in human healthy controls or LRRK2 G2019S patient-derived LCLs following a 24 h exposure of RA334 (Fig. 6B).

**Figure 7:**
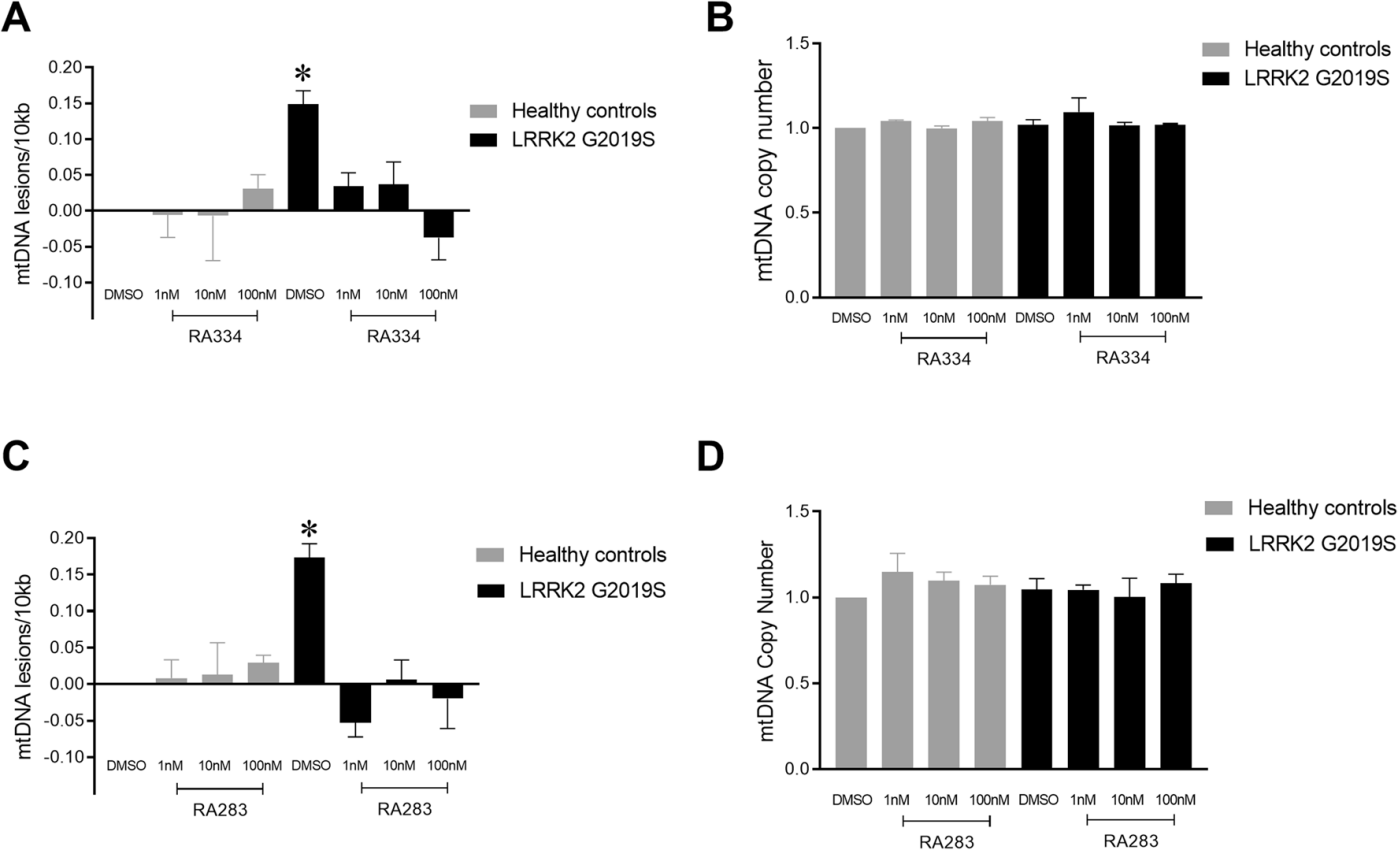
Exposure to low doses of LRRK2 kinase inhibitor also restored mtDNA damage to basal levels. Healthy control or LRRK2 G2019S patient derived LCLs were treated with either RA334 or RA283 with doses ranging from 1 to 100 nM for 24 h. (**A**) Treatment with RA334 reversed mtDNA damage in LRRK2 G2019S-patient derived LCLs, with (**B**) no effect on mtDNA copy number. (**C**) Similarly, exposure to RA283 reduced LRRK2 G2019S-induced mtDNA damage to healthy control levels. (**D**) Treatment with RA283 did not change mtDNA copy number. The PCR-based assay was performed in technical triplicate for each biological replicate. (*p < 0.001, determined by one-way ANOVA with a Tukey’s post-hoc comparison). n = 3 biological replicates (6 cell lines total), each performed in technical replicate. Data are presented as mean ± SEM.

To determine whether lower doses of RA334 or RA283 had an effect on mtDNA integrity, we next cultured control and LRRK2 G2019S patient-derived LCLs at concentrations that inhibited LRRK2 pSer935 from ~ 25 to 75% (see Figs. 2–5). Exposure of LRRK2 G2019S patient-derived LCLs to RA334 restored mtDNA damage to control levels within 24 h of exposure at all concentrations tested (Fig. 7A). No changes in mtDNA copy number were detected with exposure to RA334 (Fig. 7B). Similar results were found with 24 h treatment with RA283; 1 nM is sufficient to reverse mtDNA damage in LRRK2 G2019S-patient-derived LCLs (Fig. 7C). No differences in mtDNA copy were detected with RA283 treatment (Fig. 7D).

### LRRK2 kinase inhibition restores LRRK2 G2019S-induced mtDNA damage with acute exposure

**Figure 8:**
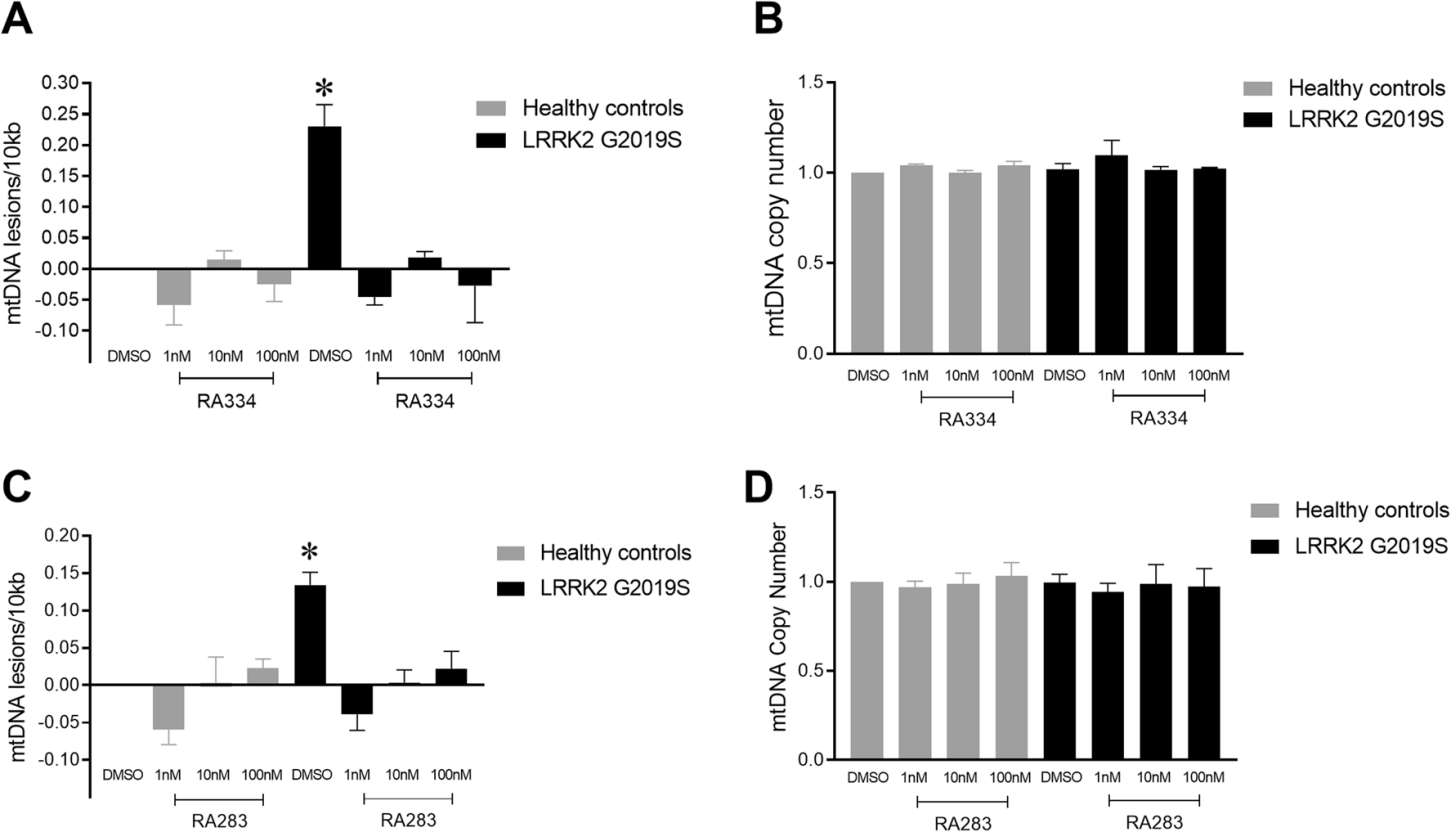
Acute exposure to LRRK2 kinase inhibitors also restored mtDNA damage to basal levels. Healthy control or LRRK2 G2019S patient derived LCLs were treated with either RA334 or RA283 with doses ranging from 1-100 nM for 1.5 h. (**A**) Treatment with RA334 reversed mtDNA damage in LRRK2 G2019S-patient derived LCLs, with (**B**) no effect on mtDNA copy number. (**C**) Similarly, exposure to RA283 reduced LRRK2 G2019S-induced mtDNA damage to healthy control levels. (**D**) Treatment with RA283 did not change mtDNA copy number. The PCR-based assay was performed in technical triplicate for each biological replicate. (*p < 0.001, determined by one-way ANOVA with a Tukey’s post-hoc comparison). n = 3 biological replicates (6 cell lines total), each performed in technical replicate. Data are presented as mean ± SEM.

The loss of constitutive phosphorylation of LRRK2 Ser935 (and Ser955, Ser973) in the presence of a LRRK2 kinase inhibitor is rapid (^38,42^, Fig. 5). To determine whether the time course of mtDNA damage reversal by LRRK2 kinase inhibitors also occurs quickly, control and LRRK2 G2019S patient-derived LCLs were exposed to RA334, RA283, or vehicle for 1.5 h. Exposure of LRRK2 G2019S patient-derived LCLs to RA334 restored mtDNA damage to control levels at concentrations ranging from 1 to 100 nM (Fig. 8A), without an effect on mtDNA copy number (Fig. 8B). Treatment with RA283 for 1.5 h similarly reversed mtDNA damage in LRRK2 G2019S-patient-derived LCLs at all concentrations tested (Fig. 8C). No differences in mtDNA copy were detected with compound RA283 acute treatment (Fig. 8D). Importantly, similar results were found with the well-studied LRRK2 kinase inhibitor MLi-2; exposure of LRRK2 G2019S patient-derived LCLs to MLi-2 restored mtDNA damage to control levels at concentrations ranging from 1 to 100 nM, without an effect on mtDNA copy number (Supplemental Fig. S16).

## Discussion

Mutations in the LRRK2 gene are the most common cause of familial PD and lead to elevated kinase activity which is thought to underlie its neurotoxic effect^4–6^. Recent studies have suggested that LRRK2 kinase activity is elevated in idiopathic PD patients without a LRRK2 mutation^43^. Given the strong link between LRRK2 and disease pathogenesis in familial and idiopathic PD, LRRK2 is a good candidate for small molecule kinase inhibitor development and highly specific inhibitors are being tested in clinical trials. An important component of a successful clinical trial is to demonstrate target engagement and understand pharmacodynamics in order to determine the best dosing for efficacy. While the majority of studies have focused on analyzing the phosphorylation of LRRK2 itself or its substrate Rab10, these readouts are either indirect, lack sensitivity or do not correlate with intrinsic cellular kinase activity of LRRK2^22–26,32^. We previously discovered that the LRRK2 G2019S mutation directly causes mtDNA damage^35^. We further demonstrated that LRRK2 G2019S-induced mtDNA damage was kinase-dependent, and LRRK2 kinase inhibition was able to abrogate mtDNA damage whether neurons were treated prior to, concurrently with or post the presence of LRRK2 G2019S-induced mtDNA damage^34^. We now extend these studies and determined whether mtDNA damage levels might serve as a useful cellular readout for LRRK2 kinase inhibition. We demonstrate that the reversal of LRRK2 G2019S-induced mtDNA damage correlates with inhibition of LRRK2 kinase activity. The observed restoration of mtDNA damage to control levels following LRRK2 kinase inhibition occurs quickly and was found at doses that blocked LRRK2 kinase activity and not with a concomitant decrease in LRRK2 protein levels. Similar to previous findings, LRRK2 Ser935 phosphorylation in healthy control and LRRK2 G2019S patient-derived LCLs with endogenous LRRK2 levels was similar in response to new and well-studied LRRK2 kinase inhibitors^36,44^. Despite the identical response of LRRK2 Ser935 dephosphorylation, there was a profound change in mtDNA damage levels in LRRK2 G2019S patient-derived LCLs and not in healthy control cells in response to multiple LRRK2 kinase inhibitors. Altogether, mtDNA damage levels are a sensitive cellular readout of altered LRRK2 kinase activity and has potential as a pharmacodynamic biomarker for utility in clinical trials with LRRK2 kinase inhibitor-targeted approaches.

The optimal therapeutic window for the efficacy of LRRK2 kinase inhibitors is unknown. There is clear evidence that too much inhibition which results in the loss of total LRRK2 protein is deleterious and mimics a homozygous LRRK2 knockout^45^. Since the near or complete loss of LRRK2 protein is associated with kidney and lung changes, the high end of the therapeutic window could be defined as inhibition levels that do not reduce total LRRK2 protein levels^16^. The two LRRK2 novel inhibitors studied here showed a modest decrease of total LRRK2 protein at concentrations at and above 10 nM, which is greater than the IC_50_. Yet how each specific tissue, for example, brain *vs* lung, might tolerate a small (< 25%) loss of total LRRK2 protein is unknown. Since antisense oligonucleotides directed towards LRRK2 are also being considered and tested as a therapy for PD, understanding the cellular consequences of decreased LRRK2 protein and the impact on kinase activity will help resolve these questions. Our results suggest that decreased LRRK2 protein was not required to observe the reversal of LRRK2 G2019S-induced mtDNA damage. It will be interesting to analyze differing levels of reduction in LRRK2 protein on mtDNA damage.

On the other hand, defining the minimum level of inhibition to achieve efficacy is more complex. However, this is crucial, because insufficient inhibition could be related to failure to observe neuroprotection in a clinical trial. The increase in kinase activity due to LRRK2 pathogenic mutations is small, thus blocking kinase activity in order to return to normal healthy levels should also be minimal. Most studies evaluating neuroprotection or effects on PD-related pathology have tested LRRK2 kinase inhibitors at high doses that reduce LRRK2 Ser935 > 90% and did not evaluate the dose-dependency of these outcomes^16^. In this study, ~ 25–45% inhibition of LRRK2 kinase activity, depending on the inhibitor, is sufficient to reverse mtDNA damage levels back to healthy control levels. This result strengthens the hypothesis that medium LRRK2 kinase inhibition is sufficient to rescue a phenotype of PD. Importantly, PD patient-derived immortalized cells were used in this study and the level of LRRK2 kinase inhibition required to observe a reversal of mtDNA damage needs to be validated in human primary blood-derived cells.

Measuring intrinsic endogenous LRRK2 kinase activity has been challenging^8,46^. While changes in mtDNA damage levels were robustly detected following LRRK2 kinase inhibition in LRRK2 G2019S patient-derived cells, this was not the case in healthy control LCLs. It is possible that wild-type LRRK2 kinase activity in control cells is either low to non-detectable, as has been reported by our group and others with measuring endogenous levels by either immunoblotting or proximity ligation assay via LRRK2 pSer1292^10,14,33,47^. Thus changes in mtDNA damage levels following LRRK2 kinase inhibition in control cells may be minimal. It is also formally possible that these changes are below the limit of detection for the PCR-based assay. Alternatively, the degree of LRRK2 kinase activity in healthy cells may be cell-type specific and will be explored in future studies.

There is substantial interest in determining whether idiopathic PD patients with altered LRRK2 activity can be identified and potentially stratified in LRRK2 kinase inhibitor trials. To date, LRRK2 or Rab10 phosphorylation show no consistent differences between healthy controls and idiopathic PD, making these biomarkers unlikely candidates for patient stratification. Recently, centrosomal cohesion alterations could be detected in both LRRK2 G2019S LCLs and a subset of idiopathic PD patient samples^48^. These cohesion deficits in subjects with idiopathic PD were reverted with a LRRK2 kinase inhibitor, despite lack of increased LRRK2 pSer1292 using a proximity ligation-based assay^48^. Centrosome alterations can be linked to genome instability and therefore mtDNA damage and cohesion deficits could be associated in the same pathway^49^. Similar to our findings with the LRRK2 G2019S mutation, we observed increased mtDNA damage in dopamine neurons from the substantia nigra in idiopathic murine models of PD and post-mortem brains derived from subjects with idiopathic PD^35^. It is not yet known whether mtDNA damage accumulation in non-familial PD is LRRK2 kinase-dependent. In a toxin rat model of PD, mtDNA damage was increased in peripheral tissue, including blood^50^. Future studies will include measurements of mtDNA damage in peripheral tissues derived from idiopathic PD patients and the response to LRRK2 kinase inhibition. There are still gaps in our knowledge regarding the mechanisms of mtDNA damage accumulation and quick reversal with blocking LRRK2 kinase activity. The speed of the reversal of LRRK2 G2019S-induced mtDNA damage highlights the dynamic nature of this phenotype, which could involve autophagy or DNA repair pathways^51–53^. While further studies into the mechanisms underlying the mitochondrial genome deficits are required, our present data indicate that mtDNA damage levels may serve as a robust and sensitive readout of altered LRRK2 kinase activity.

## Materials and methods

### Healthy subject and patient-derived lymphoblastoid cell lines

LRRK2 G2019S PD patient (n = 3) and healthy subject control (n = 3)-derived LCLs were obtained from the NINDS Coriell biorepository (cell line ID numbers and demographics are listed in Supplemental Table S1). There was not a statistically significant difference in the ages between the LRRK2 G2019S PD patient and healthy control subjects (*P* > 0.99). LCLs were cultured at 37 °C, 5% CO_2_, in RPMI-1640 (Sigma-Aldrich, R8758), 15% heat-inactivated fetal bovine serum (VWR Seradigm, 97068-091) and 0.5% Penicillin/Streptomycin (Corning, 30–002-CI). Cells were passaged every 3–4 days, and passage number did not exceed 20.

### LRRK2 kinase inhibitors

Multiple LRRK2 kinase inhibitors were utilized for in vitro experiments including MLi-2^39^, RA283 and RA334. RA283 and RA334 are two potent and selective LRRK2 inhibitors, originally discovered as part of the Sanofi chemistry program to identify inhibitor compounds targeting the LRRK2 kinase domain. Using the LanthaScreen kinase assay, RA283 and RA334 inhibited G2019S mutant LRRK2 kinase activity with an IC_50_ value of 5 and 2 nM, respectively, with 1.34 mM ATP (10 km). In stably transfected HEK293 cells with LRRK2 G2019S, LRRK2 pSer935 IC_50_ was 5 nM for RA283 and 6 nM for RA334. The kinase selectivity of both compounds was assessed using a Eurofin panel of 315 kinases. RA283 hit only 2 off-targets with IC_50_ below 500 nM at 1 km ATP (MLK1: 108 nM; ACK1: 188 nM). Under the same conditions, RA334 hit 2 off-targets (JNK3: 58 nM; MLK1: 125 nM). For all drug treatments, cells were treated for 24 or 1.5 h with varying doses of Compound RA283, RA334 or MLi-2 dissolved in DMSO. Final DMSO concentrations in in vitro cellular treatments did not exceed 0.1% v/v. The structure of RA283 and RA334 cannot be disclosed at this stage. The structure will be published in an upcoming publication.

### Cell viability and apoptosis measurements

Both healthy control and LRRK2 G2019S PD patient-derived LCLs were stained with Annexin V conjugated to a FITC dye and propidium iodide per manufacturer instructions using an Annexin V/PI kit (Thermo Fisher, V13241). Classifications were as follows: cells positive for propidium iodide (with or without Annexin V) were considered dead, cells only positive for Annexin V were apoptotic, and cells negative for both dyes were considered live. Within 30 min of staining, cells were analyzed via flow cytometry using a BD FACSCanto system, and at least 10,000 events were recorded per sample. Data analysis was performed using FlowJo version 10.6.1.

### Western blot analysis

Five million cells were pelleted and resuspended in 100 µl of lysis buffer consisting of 1% Triton X-100, 50 mM Trizma-HCl, 150 mM NaCl, 1 mM EDTA, protease inhibitor cocktail (Sigma-Aldrich, P8340), and Halt phosphatase inhibitor cocktail (Thermo Fisher, 78420). After a 10-min incubation on ice, lysates were spun at 10,000×*g* and the supernatant was collected. The protein was quantified using the DC protein assay (Bio-Rad, 5000112). Due to differing endogenous LRRK2 levels, 40 µg of ND2559, 60 µg of ND264, or 100 µg of ND11, ND1618, ND312, ND2752 sample were incubated at 100 °C for 5 min with NuPAGE Sample loading dye (Thermo Fisher, NP0007) and dithiothreitol as reducing agent. After SDS-PAGE, the blots were blocked in 5% w/v nonfat dry milk in 1X PBST (0.05% Tween 20). For our investigations, the following primary antibodies were used: rabbit anti-LRRK2 c41-2 (Abcam, ab133474, 1:2000), mouse anti-LRRK2 N241A/34 (Antibodies Inc., 75-253, 1:2000), rabbit anti-LRRK2 pS935 (Abcam, ab133450, 1:2000), rabbit anti-LRRK2 pS955 (Abcam, ab169521, 1:2000), rabbit anti-LRRK2 pS973 (Abcam, ab181364, 1:2000), mouse anti-Rab10 (Abcam, ab104859, 1:1000), rabbit anti-Rab10 pT73 (Abcam, ab230261, 1:1000), mouse anti-Rab8a (Novus, H00004218-M02, 1:2000), rabbit anti-Rab8a pT72 (Abcam, ab230260, 1:1000), mouse anti-β-actin (Novus, 8H10D10, 1:10,000). The blots were then probed with fluorescent-labeled secondary antibodies, IRDye donkey anti-mouse and anti-rabbit at 1:10,000 (LI-COR, 926-32212, 926-32213, 926-68072, 926-68073), and scanned using an Odyssey Imaging scanner (LI-COR). Fluorescent intensities were quantified using ImageStudio Lite software (LI-COR), and the signal from the protein of interest was normalized to the fluorescent intensity of either LRRK2 or β-actin. Values were averaged from at least three technical replicates within a cell line, and three biological replicates (three control lines and three PD cell lines). Of note, we attempted to evaluate LRRK2 Ser910 levels (Abcam, ab133449), yet this was not feasible due to a plethora of non-specific cross-reacting bands. We also attempted to measure LRRK2 Ser1292 levels by western blot using the commercially available antibody (Abcam ab203181). Using standard methods, we were unable to detect endogenous levels of LRRK2 Ser1292 in either controls or LRRK2 G2019S patient-derived LCLs.

### DNA isolation and quantitation

Cells were collected and the nuclei and mitochondria were isolated as previously described^35,54^. DNA was extracted with either the QuickGene DNA Whole Blood Kit L (Autogen, fk-dbl) utilizing a semi-automated system (Autogen, QuickGene-610L) or with the Genomic-tip 20/G kit (QIAGEN, 10223). For the Autogen system, the nuclei/mitochondria pellet was resuspended in 2 ml of 1X PBS, and the standard manufacturer’s protocol was conducted as if the cell suspension was whole blood. For DNA extraction using the Genomic-tip kit, the protocol was performed as previously described^34,35,54^. DNA was eluted with EDTA-free buffer, and quality was assessed using a Spectradrop microvolume microplate (Molecular Devices). Double-stranded DNA was quantified using Quant-iT Picogreen dsDNA assay (Thermo Fisher) as previously described^55,56^. Due to the fact that the Autogen automated system is much gentler on the DNA during extraction, it was necessary to freeze–thaw the DNA sample 3 times before PCR analysis to reduce mtDNA supercoiling.

### PCR-based mitochondrial DNA damage assay

DNA damage in the mitochondrial genome was measured utilizing a PCR-based assay, currently the most robust way of measuring damage in mtDNA^57^. This assay to calculate mitochondrial DNA lesion frequency was performed as previously described^34,55,56^. Briefly, 15 ng of DNA was used to amplify long or short amplicons of the mitochondrial genome (as determined by primer sets). The amount of amplification is directly proportional to the number of undamaged DNA templates. Average lesion frequency is calculated as -lnAD/AO, where AD is the amplification of the damaged or experimental template and AO is the amplification of the undamaged or control template. Results are then presented as lesions per 10 kb, with the values in experimental samples normalized to control samples. PCR reactions included KAPA Long Range HotStart DNA Polymerase (KAPA Biosystems) in a 96-well platform^58^. Primers used can be found in Santos, et al.^59^. Each biological DNA sample was analyzed in technical triplicate.

### Statistical analyses

Data were analyzed in Prism 8 software (GraphPad). Data were analyzed by either unpaired, two-tailed Student t-test or ANOVA with Tukey’s post-hoc analysis. *P*-values < 0.05 were considered significant. For all graphs, the bars represent mean ± standard error of the mean (SEM).

## Supplementary information

Supplementary Information.

## Data Availability

Original western blots presented in the figures are included in the Supplementary Data. All relevant data, materials, and protocols are available upon request.
