# Supplementary material for: Mitochondrial DNA damage as a potential biomarker of LRRK2 kinase activity in LRRK2 Parkinson’s disease
Source: Sci Rep. 2020 Oct 14;10:17293. doi: 10.1038/s41598-020-74195-6 (PMC7557909; doi:10.1038/s41598-020-74195-6)
Supplement: Supplementary file 1 — Supplementary Information. [file 41598_2020_74195_MOESM1_ESM.pdf]

Mitochondrial DNA damage as a potential biomarker of LRRK2 kinase activity in LRRK2 Parkinson's disease

Supplementary Information

Gonzalez-Hunt, C.P.[1], Thacker, E.A.[1], Toste, C.M.[1], Boularand, S.[2], Deprets, S. [2], Dubois, L.[2], and Sanders, L.H.[1*]

[1]Department of Neurology, Duke University Medical Center, Durham, NC 27710
[2] Rare & Neurologic Diseases Research, Sanofi, Chilly Mazarin, France

*To whom correspondence should be addressed: Dr. Laurie Sanders, Telephone: 919-613-3890, Email: laurie.sanders@duke.edu

Cell line ID #	Age at biopsy	Genetic mutation	Clinical Status	Sex
**ND02559**	46	None detected	Unaffected	M
**ND01618**	46	LRRK2 G2019S heterozygous	Affected	M
**ND00312**	72	None detected	Unaffected	M
**ND02752**	72	LRRK2 G2019S heterozygous	Affected	M
**ND00011**	75	None detected	Unaffected	F
**ND00264**	75	LRRK2 G2019S heterozygous	Affected	F

Supplemental Table 1. Lymphoblastoid cell lines: ID numbers, individual age at biopsy, genotypes, clinical status, and sex.

Assay	IC_{50} (nM)			
	RA283	RA334	GNE-7915	MLi-2
**Biochemical LRRK2 G2019S kinase assay (LanthaScreen)**	5	2	7	2
**pSer935 (G2019S HEK293)**	15	7	45	2
**Off-target kinases (< 500nM)**	MLK1: 108 nM<br>ACK1: 188 nM	MLK1: 125 nM<br>JNK3: 58 nM	-	-

Supplemental Table 2. Additional information for novel LRRK2 kinase inhibitors RA283 and RA334. Data from LanthaScreen kinase assay using 1.1 mM ATP, dephosphorylation of pSer935 in stably transfected LRRK2 G2019S HEK293 cells, and eurofin panel of 315 kinases for selectivity at 1 Km ATP.

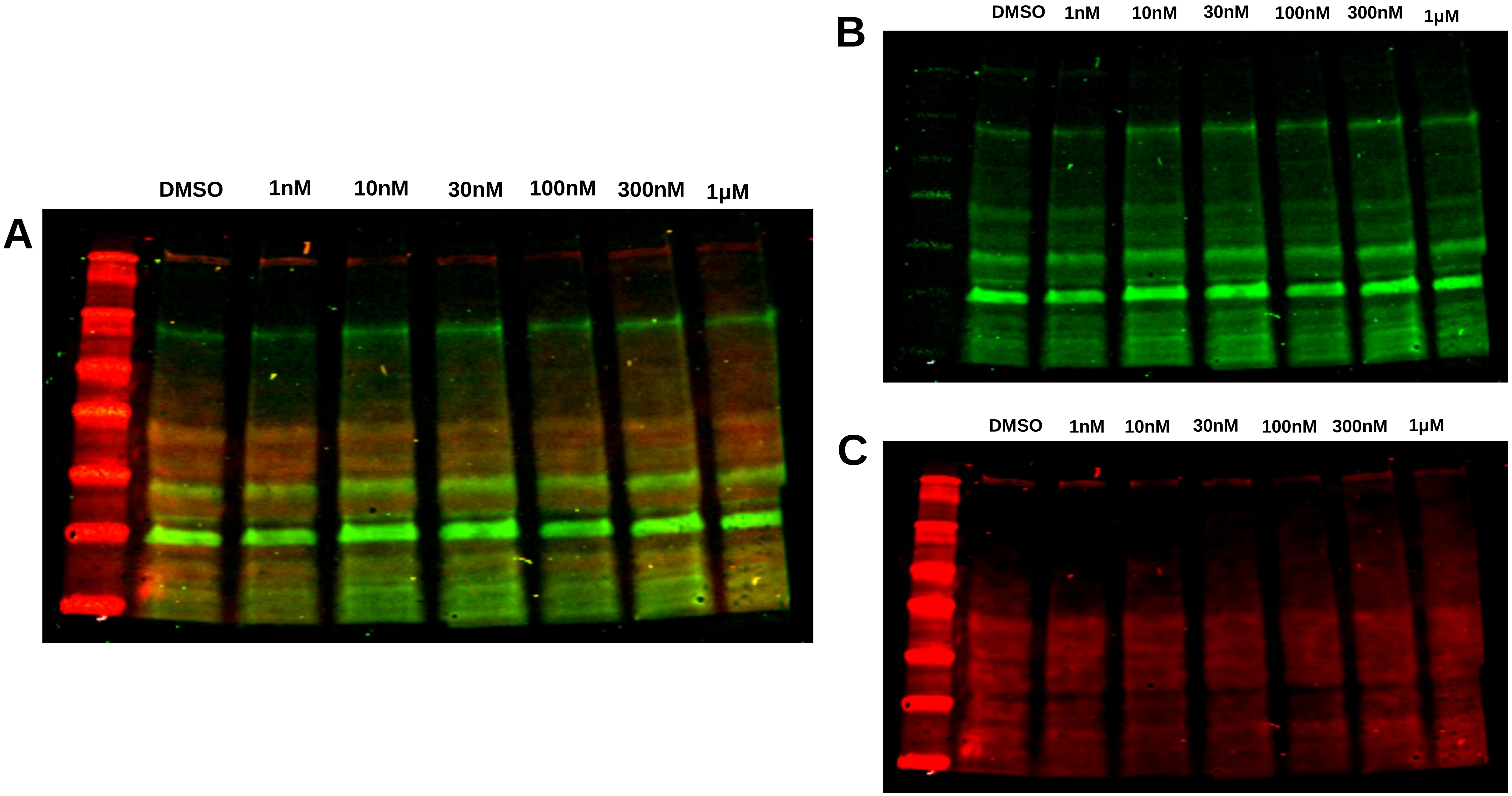

Supplemental Figure S1. Whole western blot for control samples against LRRK2 pSer955 after 24h treatment with RA283. A) Merged image of 700 nm and 800 nm channels acquired on a LI-COR Odyssey imaging system. B) 800 nm channel depicting LRRK2 pSer955. C) 700 nm channel depicting total LRRK2.

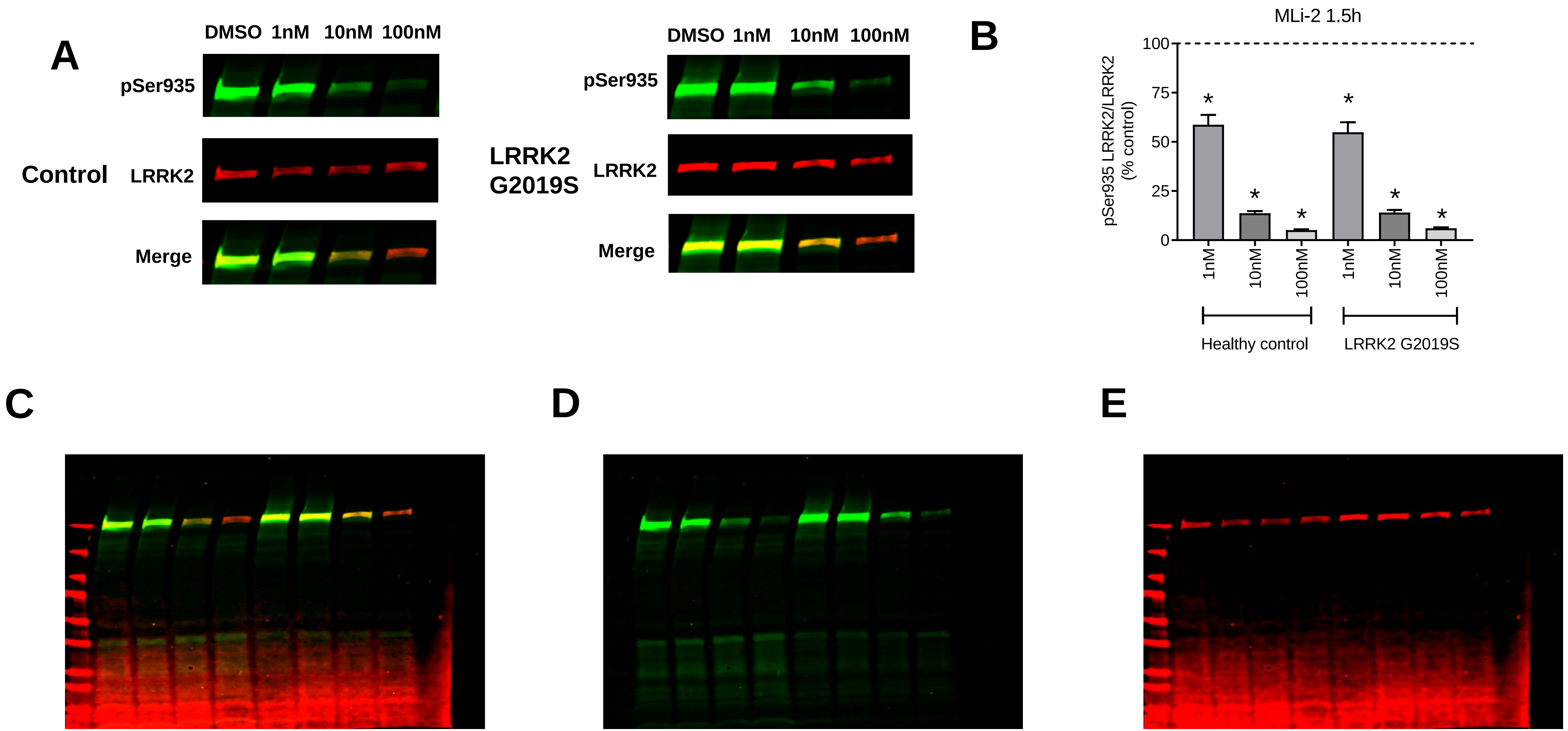

Supplemental Figure S2. Acute LRRK2 kinase inhibition with MLi-2. A) Representative western blot of healthy control and LRRK2 G2019S patient-derived LCLs treated for 1.5h with MLi-2. (B) Quantification of western blots demonstrating that MLi-2 decreased LRRK2 pSer935 levels at all doses tested. Data are mean ± SEM (*p<0.001, determined by one-way ANOVA with Tukey's post-hoc comparison). n=3 biological replicates (6 cell lines total). C) Full merged image of 700 nm and 800 nm channels for western blot in A, acquired on a LI-COR Odyssey imaging system. D) 800 nm channel depicting pSer935 LRRK2. E) 700 nm channel depicting total LRRK2.

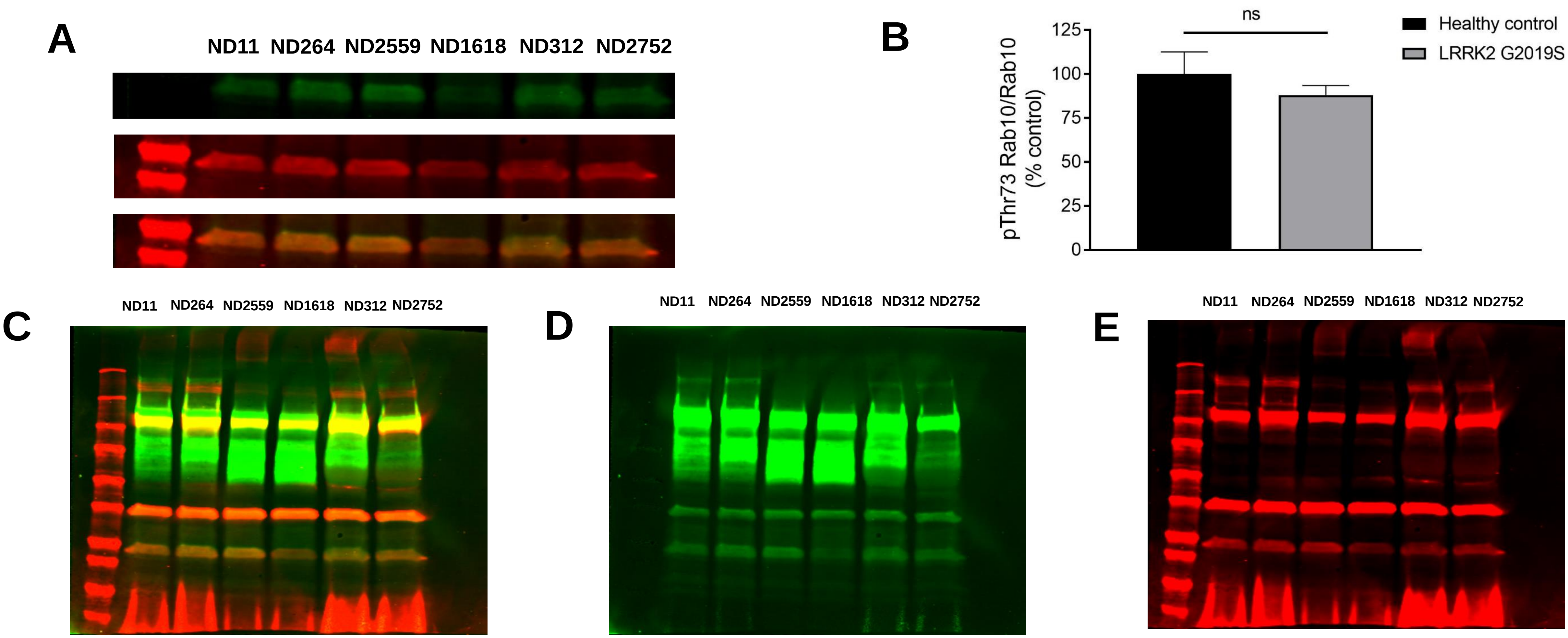

Supplemental Figure S3. pThr73 Rab10 basal levels in lymphoblastoid cell lines. A) Representative western blot of healthy control and LRRK2 G2019S patient-derived LCLs. B) Quantification of western blot in A. Data are mean ± SEM ($p=0.4308$, determined by unpaired, two-tailed student's t-test). n=3 biological replicates (6 cell lines total). C) Full merged image of 700 nm and 800 nm channels for western blot in A, acquired on a LI-COR Odyssey imaging system. D) 800 nm channel depicting pThr73 Rab10. E) 700 nm channel depicting total Rab10.

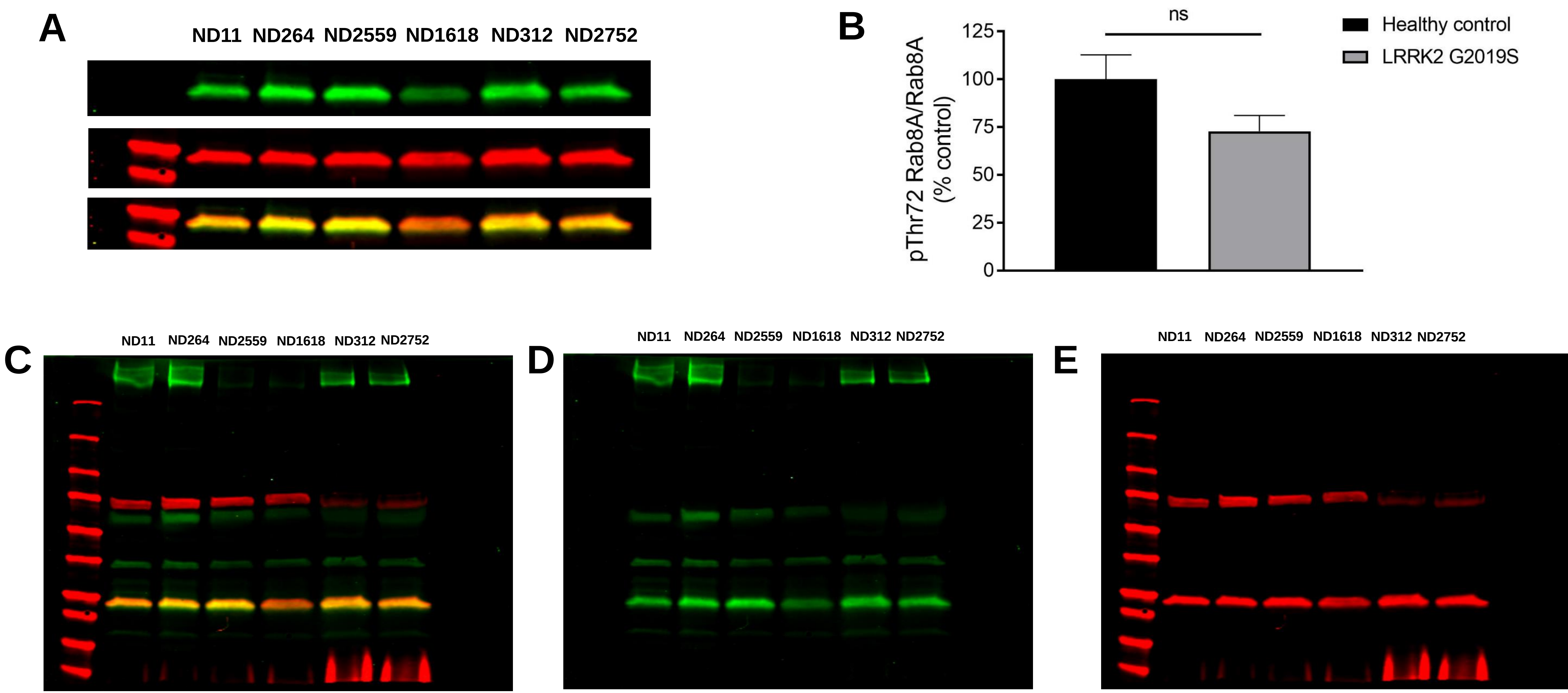

Supplemental Figure S4. pThr72 Rab8A basal levels in lymphoblastoid cell lines. A) Representative western blot of healthy control and LRRK2 G2019S patient-derived LCLs. B) Quantification of western blot in A. Data are mean ± SEM. (p=0.1487, determined by unpaired, two-tailed student's t-test). n=3 biological replicates (6 cell lines total). C) Full merged image of 700 nm and 800 nm channels for western blot in A, acquired on a LI-COR Odyssey imaging system. D) 800 nm channel depicting pThr72 Rab8A. E) 700 nm channel depicting total Rab8A.

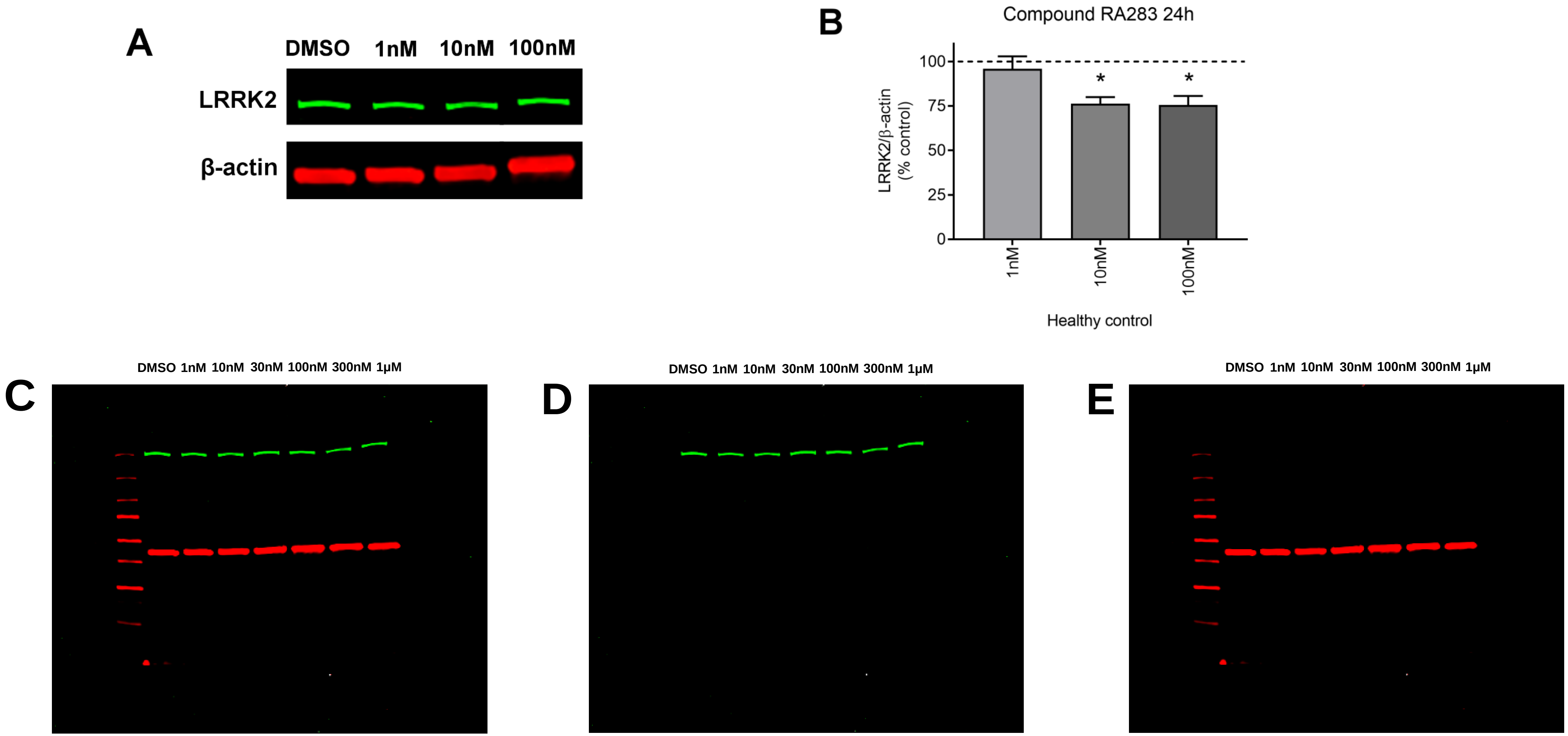

Supplemental Figure S5. Total LRRK2 levels following LRRK2 kinase inhibition. A) Representative western blots of healthy control LCLs treated for 24h with Compound RA283. B) Quantification of the western blots demonstrate that RA283 decreased LRRK2 at 10nM and above. C) Merged image of 700 nm and 800 nm channels acquired on a LI-COR Odyssey imaging system. D) 800 nm channel depicting total LRRK2. E) 700 nm channel depicting β-actin. All experiments were performed with at least three biological replicates. Data are presented as mean ± SEM. (*p <0.05, determined by one-way ANOVA with a Tukey's posthoc comparison).

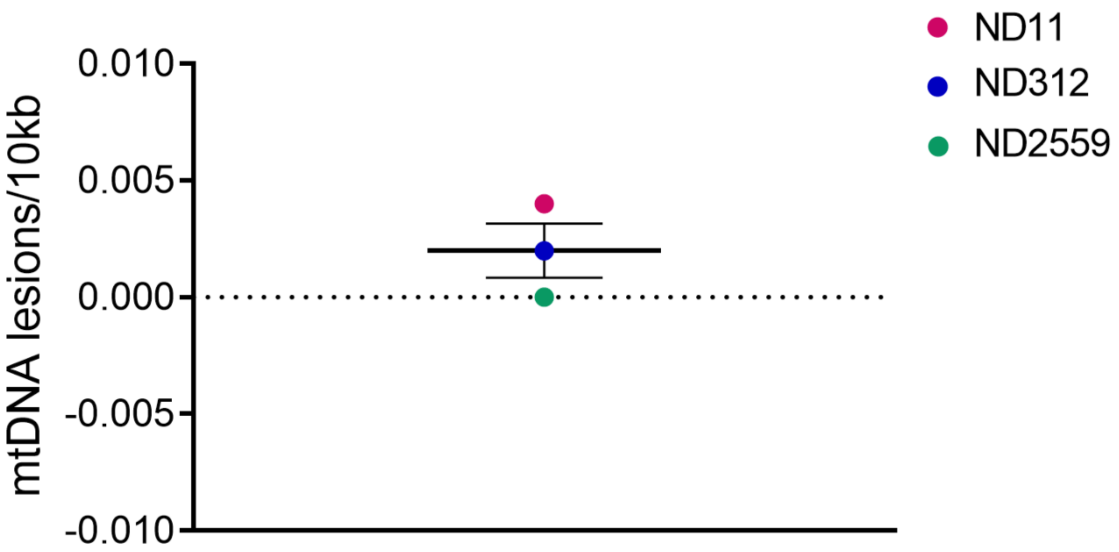

Supplemental Figure S6. mtDNA damage levels in healthy control lymphoblastoid cell lines utilized in this study. Damage levels normalized to ND2559 cell line.

Supplemental Figure S7. Whole western blot for control samples from Figure 2. For the main figure, image editing software was used to crop the line of bands for clarity. A) Merged image of 700 nm and 800 nm channels acquired on a LI-COR Odyssey imaging system. B) 800 nm channel depicting LRRK2 pSer935. C) 700 nm channel depicting total LRRK2.

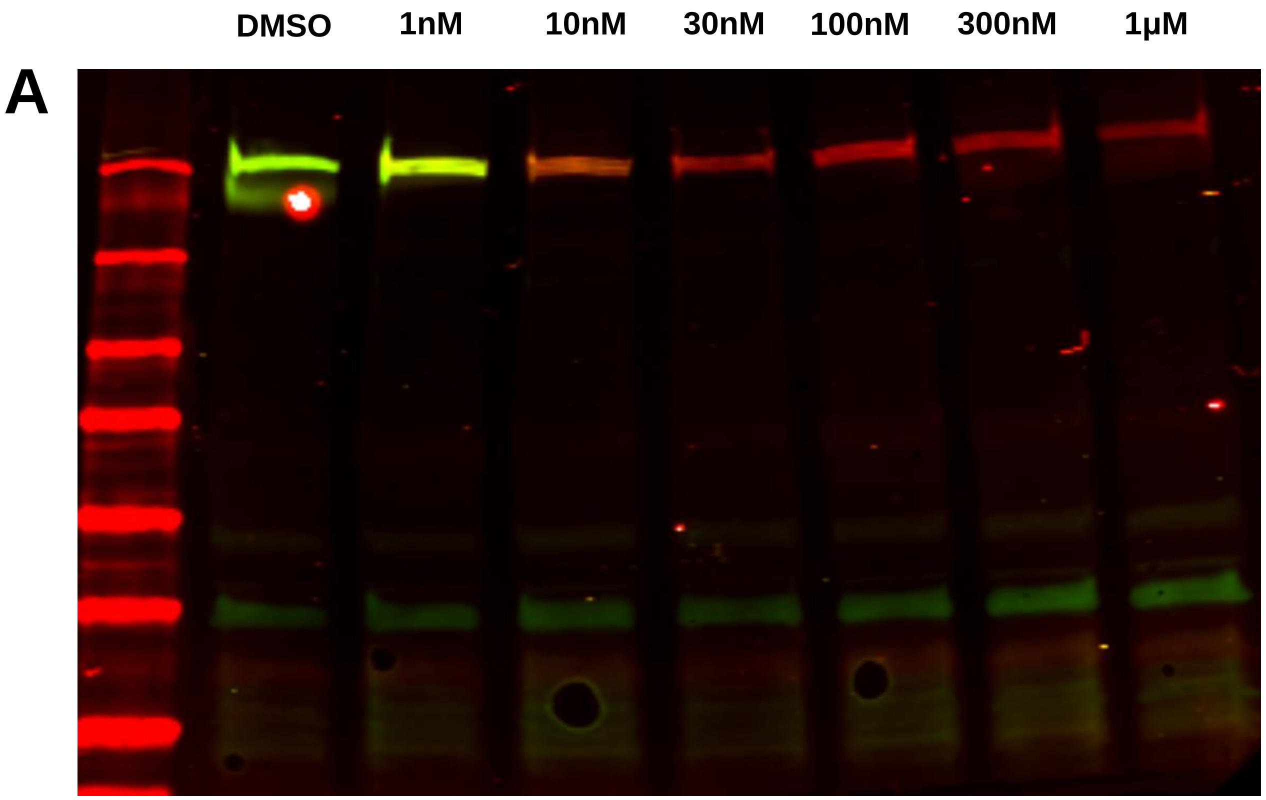

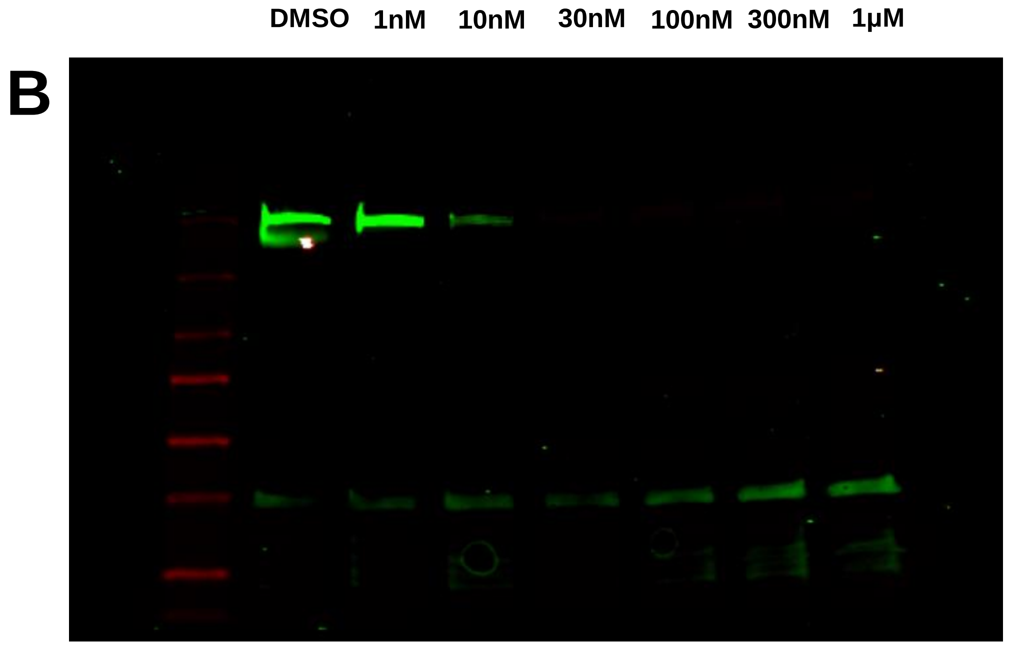

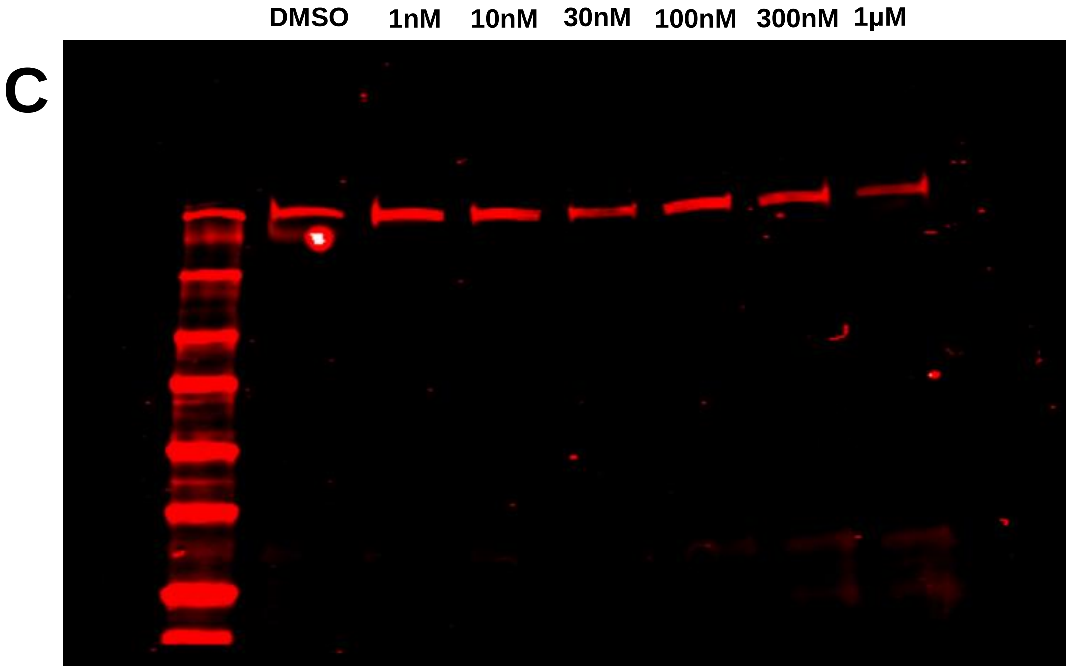

Supplemental Figure S8. Whole western blot for LRRK2 G2019S samples from Figure 2. For the main figure, image editing software was used to crop the line of bands for clarity. A) Merged image of 700 nm and 800 nm channels acquired on a LI-COR Odyssey imaging system. B) 800 nm channel depicting LRRK2 pSer935. C) 700 nm channel depicting total LRRK2.

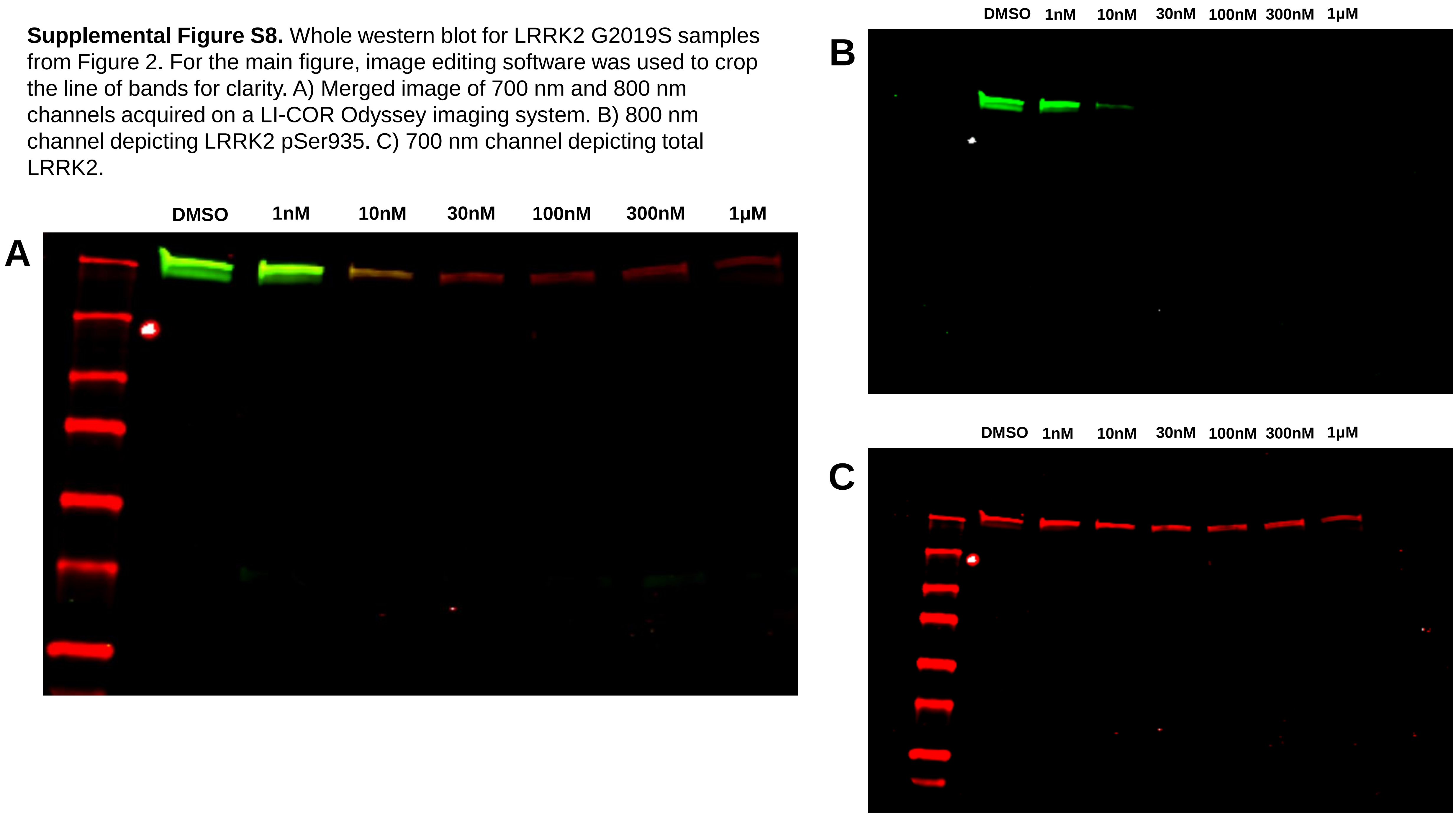

Supplemental Figure S9. Whole western blot for control samples from Figure 3. For the main figure, image editing software was used to crop the line of bands for clarity. A) Merged image of 700 nm and 800 nm channels acquired on a LI-COR Odyssey imaging system. B) 800 nm channel depicting LRRK2 pSer935. C) 700 nm channel depicting total LRRK2.

A

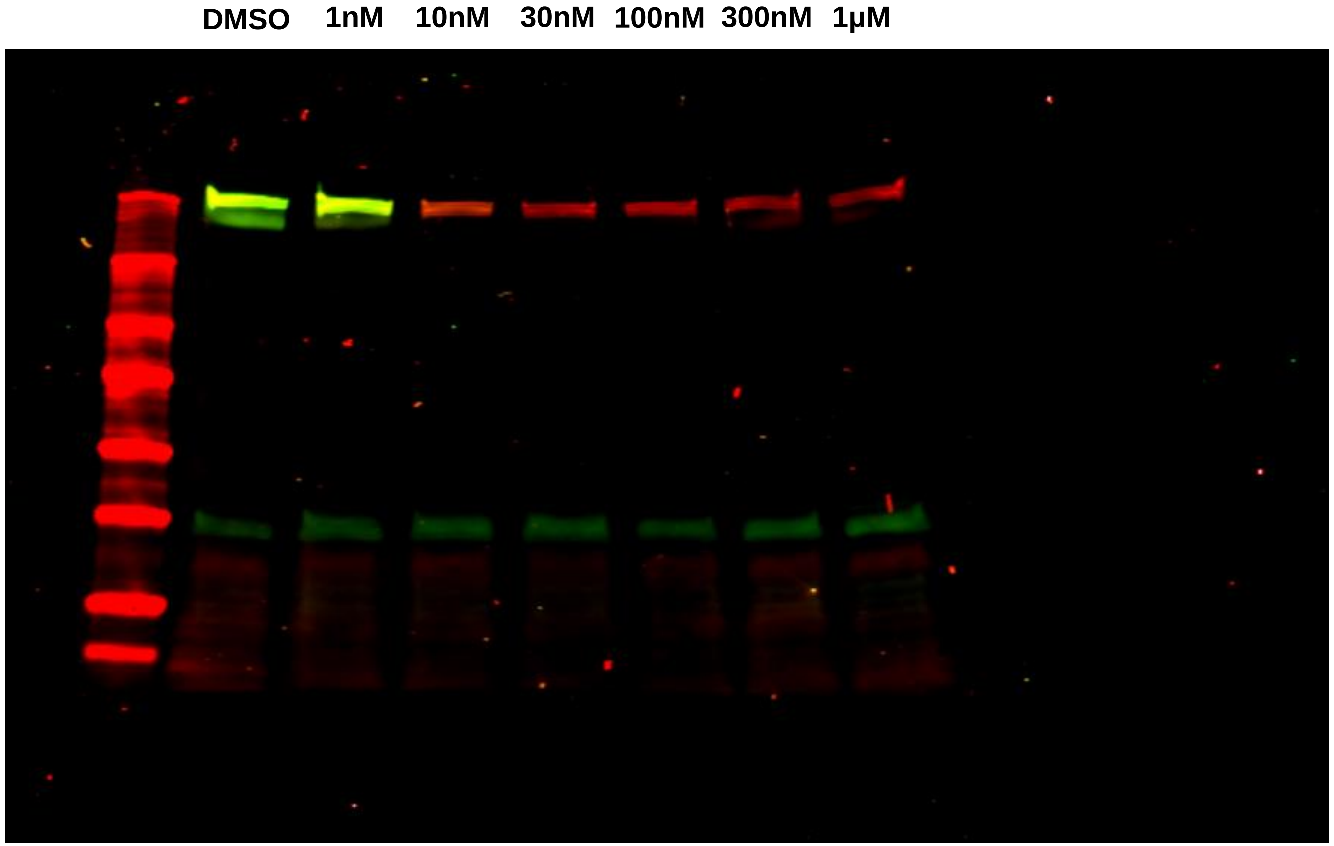

B

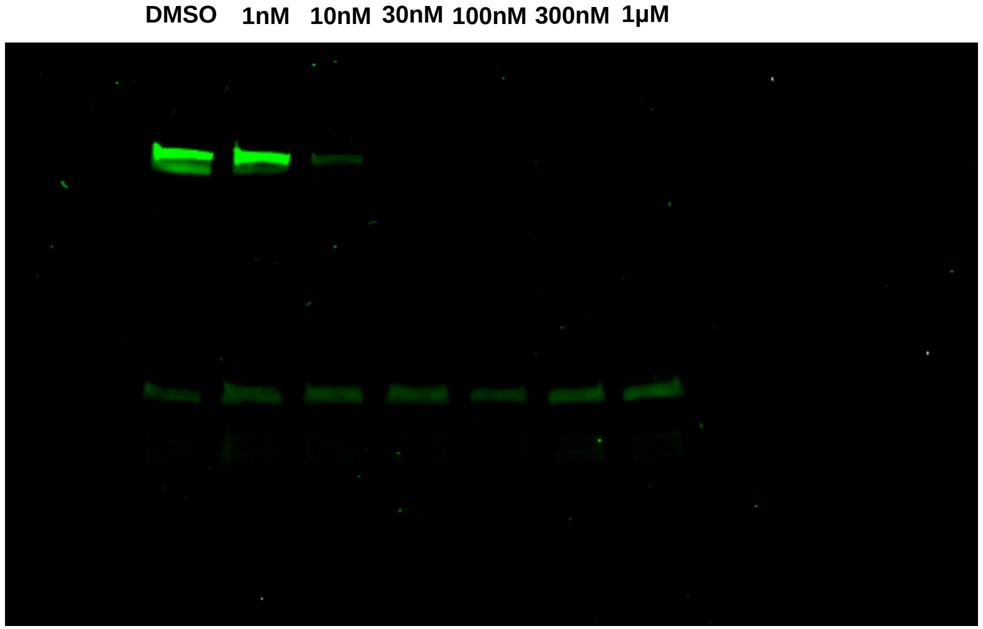

C

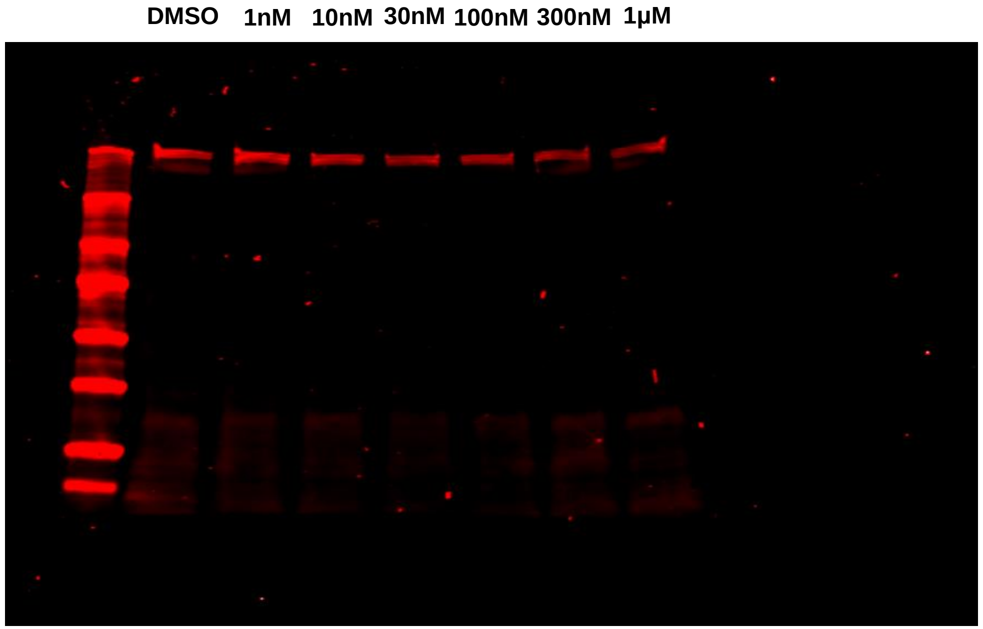

Supplemental Figure S10. Whole western blot for LRRK2 G2019S samples from Figure 3. For the main figure, image editing software was used to crop the line of bands for clarity. A) Merged image of 700 nm and 800 nm channels acquired on a LI-COR Odyssey imaging system. B) 800 nm channel depicting LRRK2 pSer935. C) 700 nm channel depicting total LRRK2.

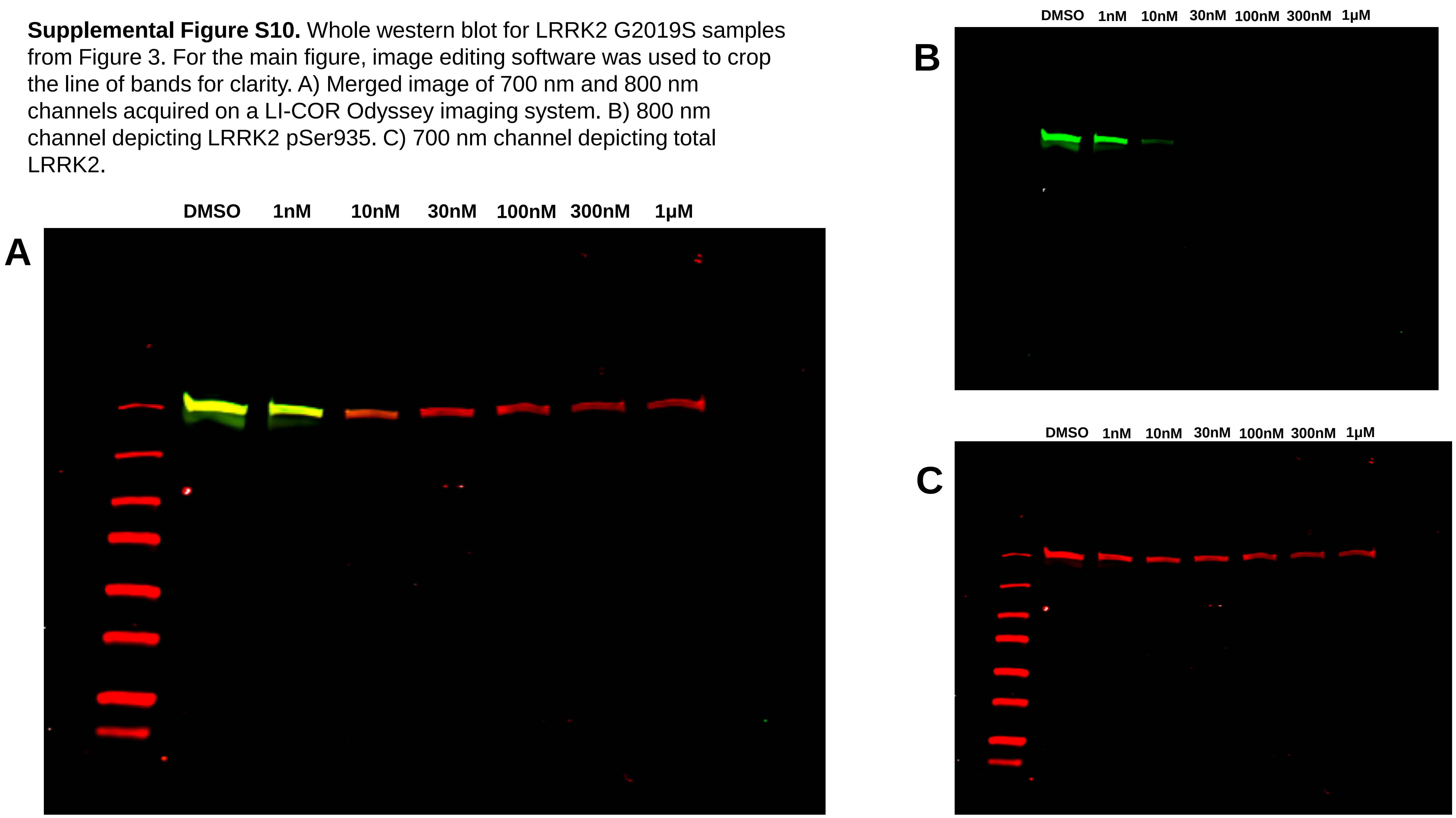

Supplemental Figure S11. Whole western blot for LRRK2 G2019S samples from Figure 4, Panel A. For the main figure, image editing software was used to crop the line of bands for clarity, and to cut the blot in half as only one experimental rep was used to create the figure. A) Merged image of 700 nm and 800 nm channels acquired on a LI-COR Odyssey imaging system. B) 800 nm channel depicting LRRK2 pSer955. C) 700 nm channel depicting total LRRK2.

DMSO 1nM 10nM 100nM DMSO 1nM 10nM 100nM

A

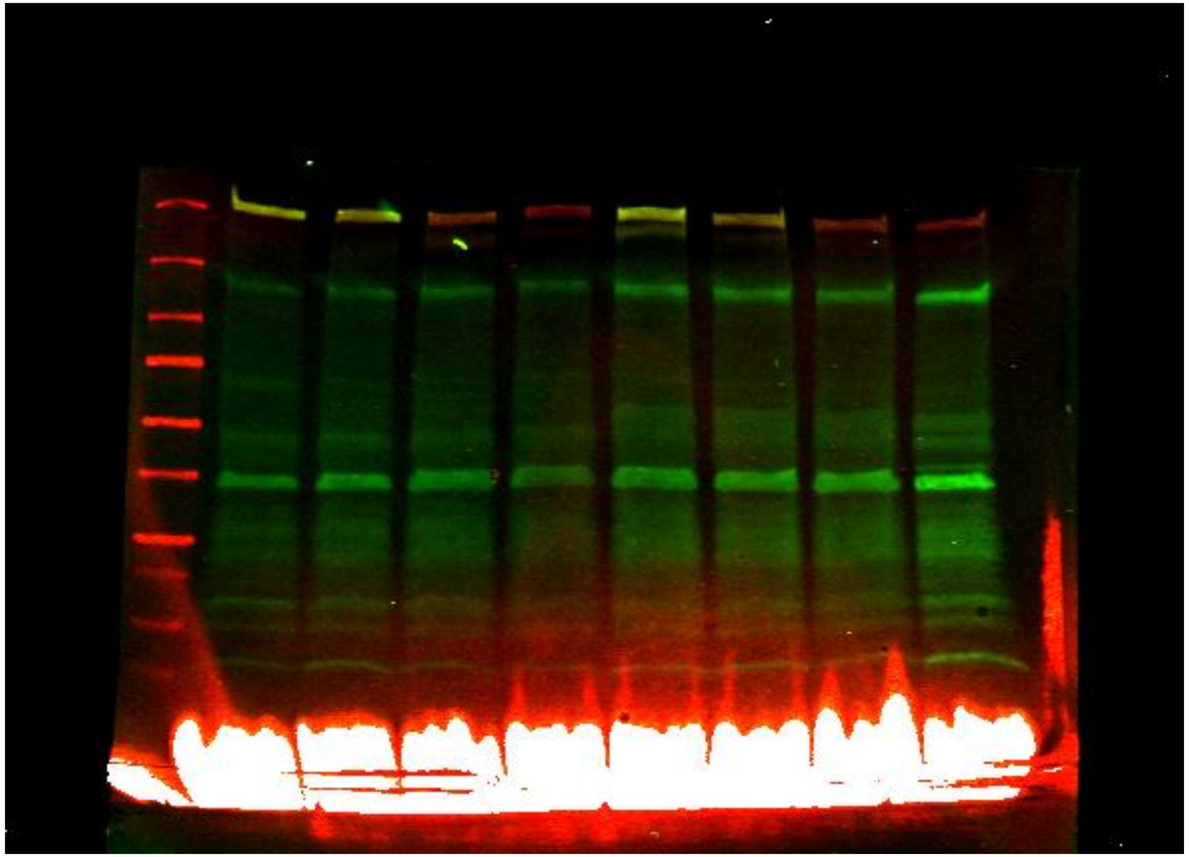

DMSO 1nM 10nM 100nM DMSO 1nM 10nM 100nM

B

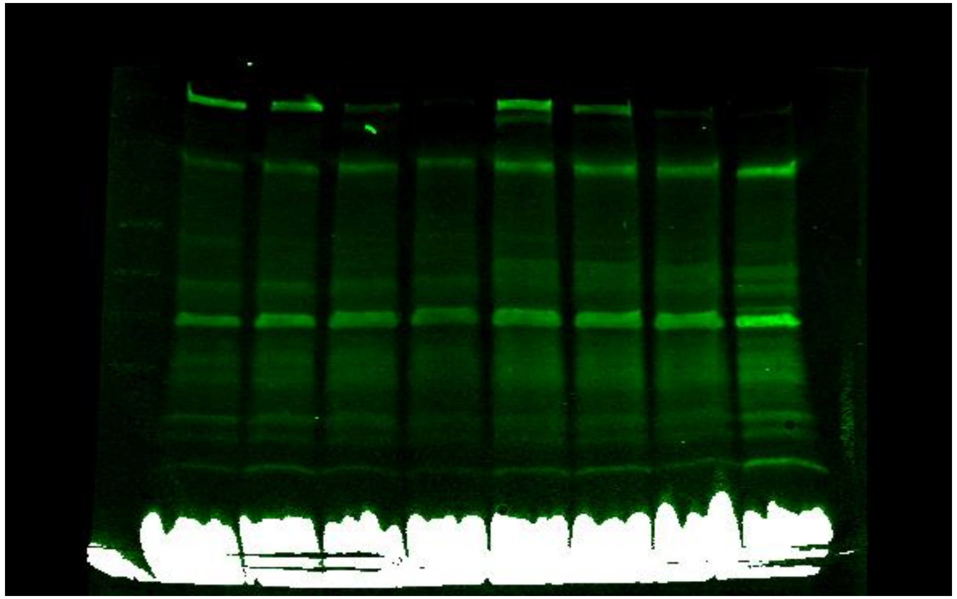

DMSO 1nM 10nM 100nM DMSO 1nM 10nM 100nM

C

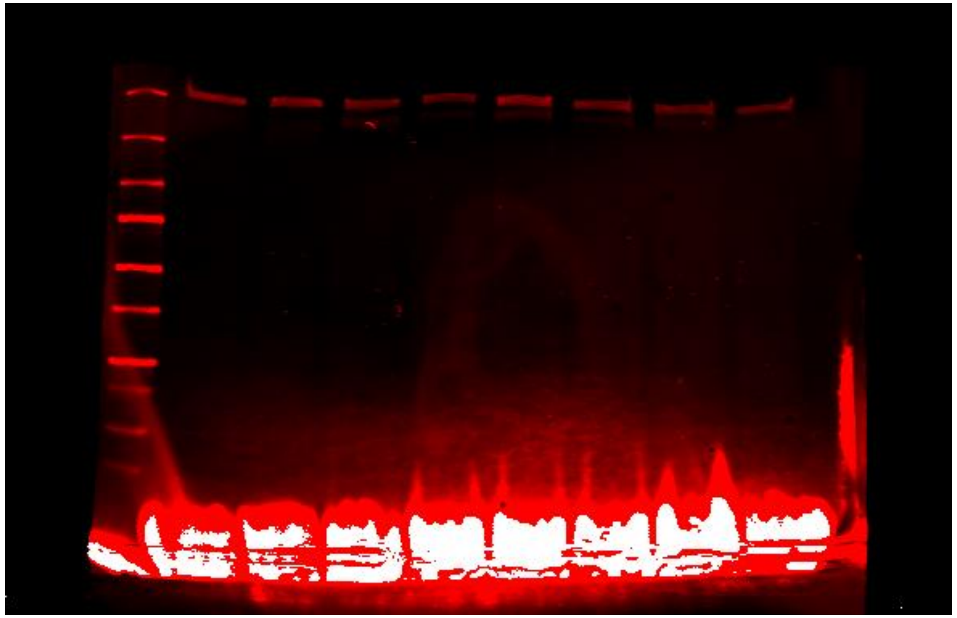

Supplemental Figure S12. Whole western blot for control samples from Figure 4, Panel C. For the main figure, image editing software was used to crop the line of bands for clarity, and to cut and splice together the relevant doses of RA283. A) Merged image of 700 nm and 800 nm channels acquired on a LI-COR Odyssey imaging system. B) 800 nm channel depicting LRRK2 pSer973. C) 700 nm channel depicting total LRRK2.

A

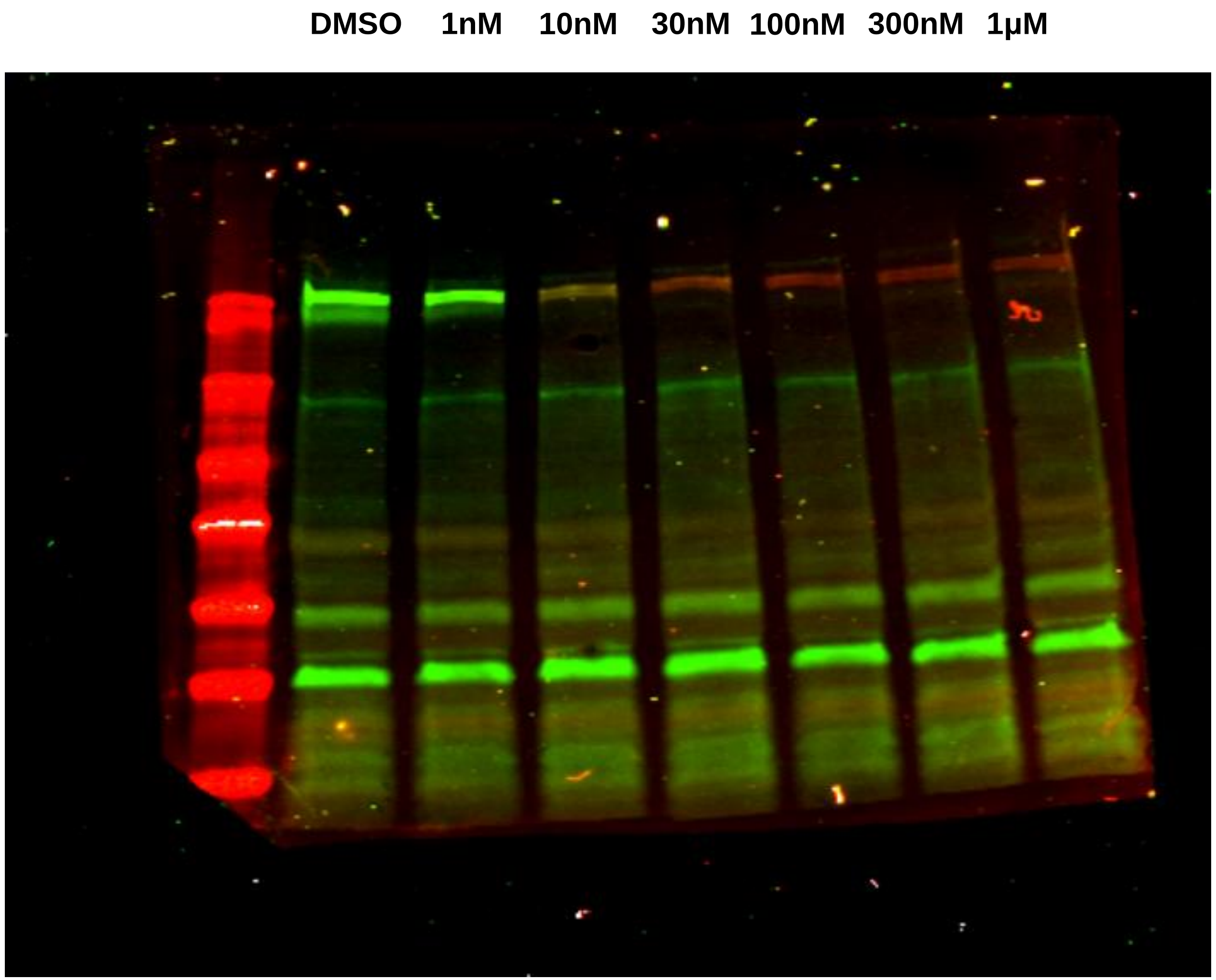

B

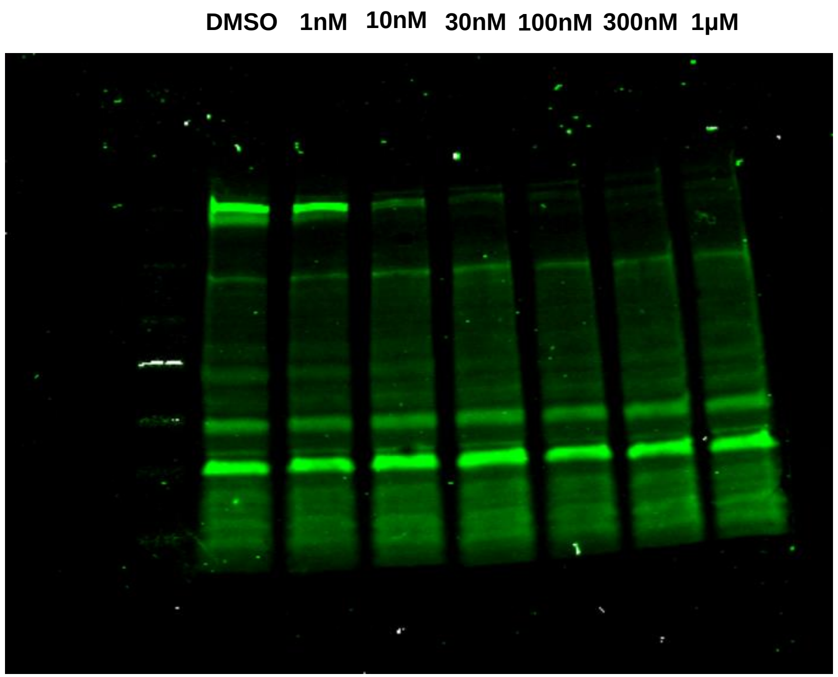

C

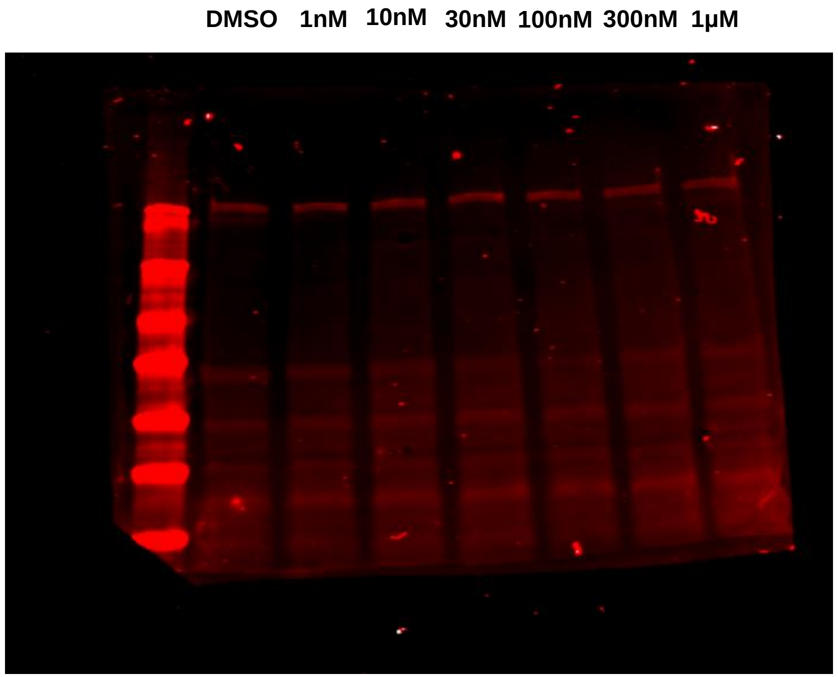

Supplemental Figure S13. Whole western blot for LRRK2 G2019S samples from Figure 4, Panel C. For the main figure, image editing software was used to crop the line of bands for clarity, and to cut and splice together the relevant doses of RA283. A) Merged image of 700 nm and 800 nm channels acquired on a LI-COR Odyssey imaging system. B) 800 nm channel depicting LRRK2 pSer973. C) 700 nm channel depicting total LRRK2.

A

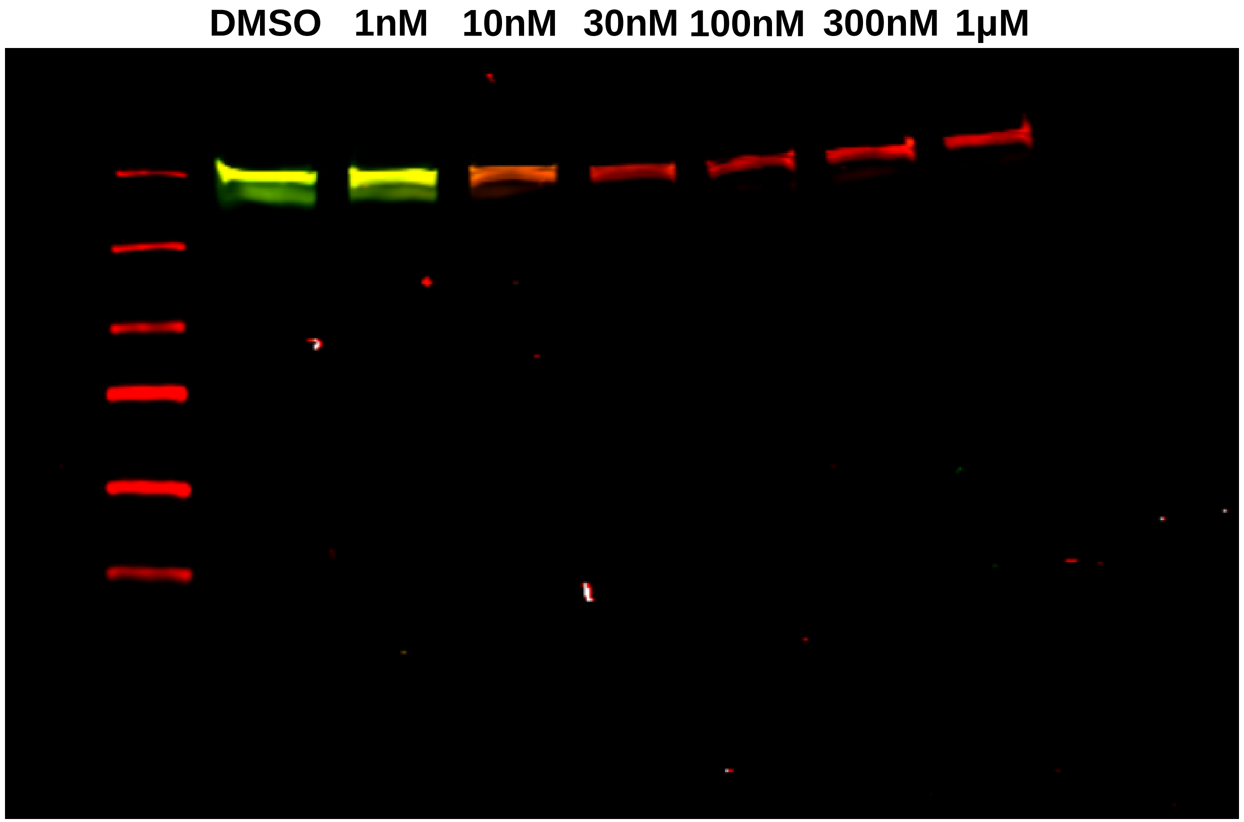

B

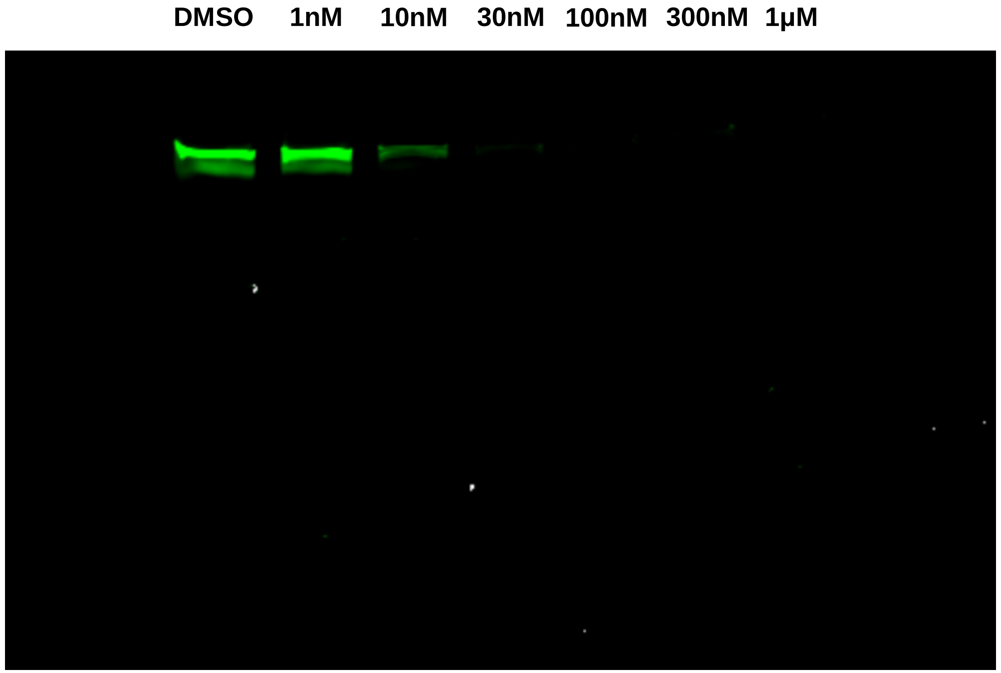

C

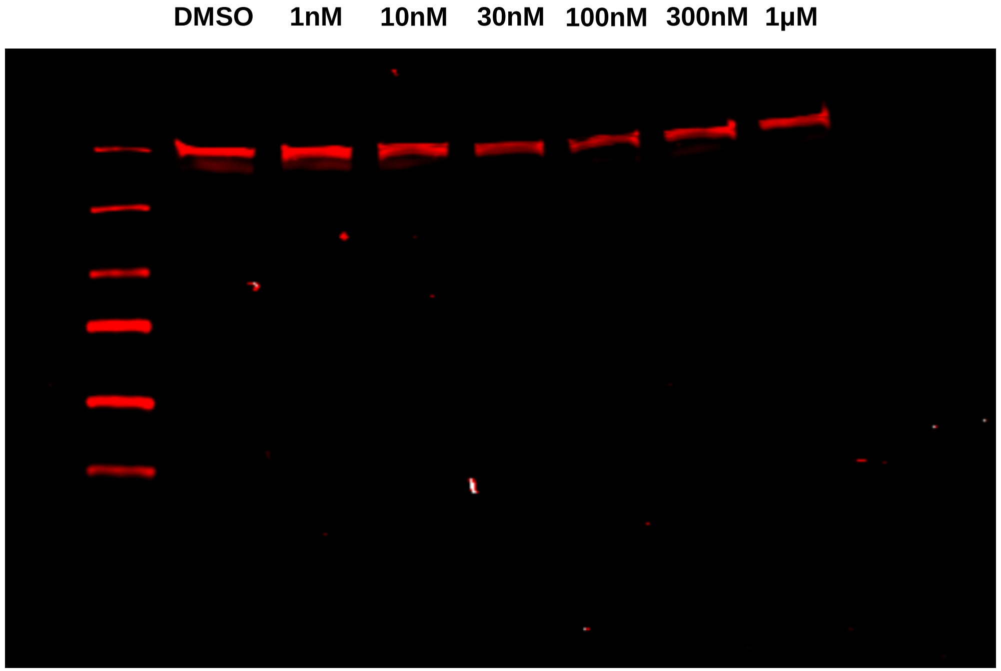

Supplemental Figure S14. Whole western blot for control and LRRK2 G2019S samples from Figure 5, Panel A. For the main figure, image editing software was used to crop the line of bands for clarity, and to cut the blot in half. A) Merged image of 700 nm and 800 nm channels acquired on a LI-COR Odyssey imaging system. B) 800 nm channel depicting LRRK2 pSer935. C) 700 nm channel depicting total LRRK2.

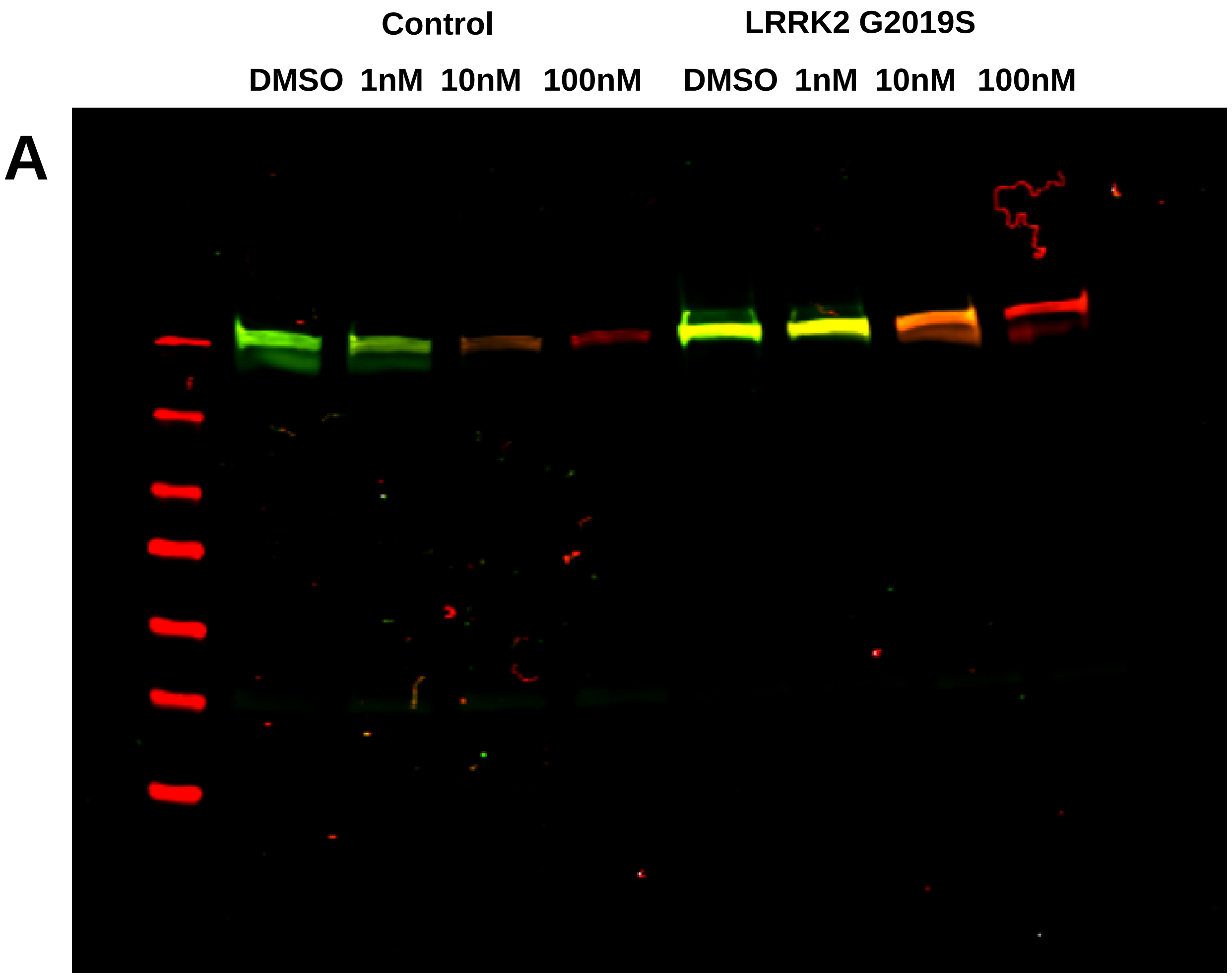

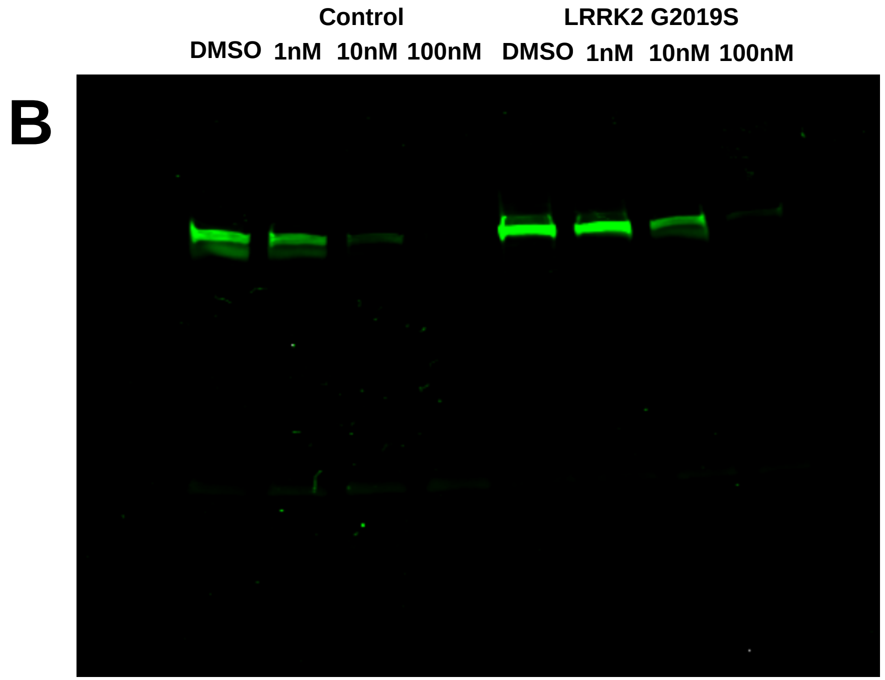

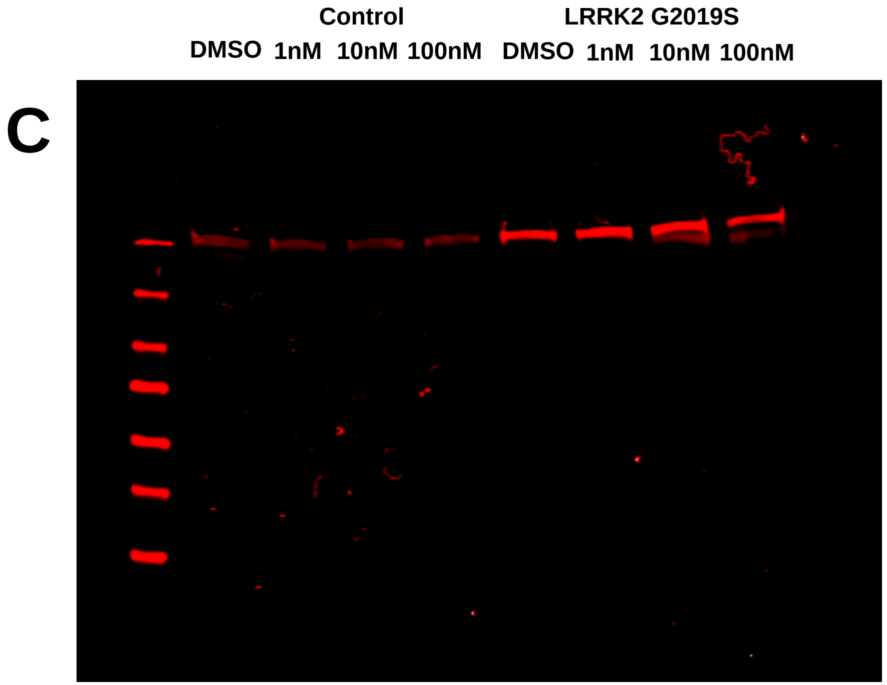

Supplemental Figure S15. Whole western blot for control and LRRK2 G2019S samples from Figure 5, Panel C. For the main figure, image editing software was used to crop the line of bands for clarity, and to cut the blot in half. A) Merged image of 700 nm and 800 nm channels acquired on a LI-COR Odyssey imaging system. B) 800 nm channel depicting LRRK2 pSer935. C) 700 nm channel depicting total LRRK2.

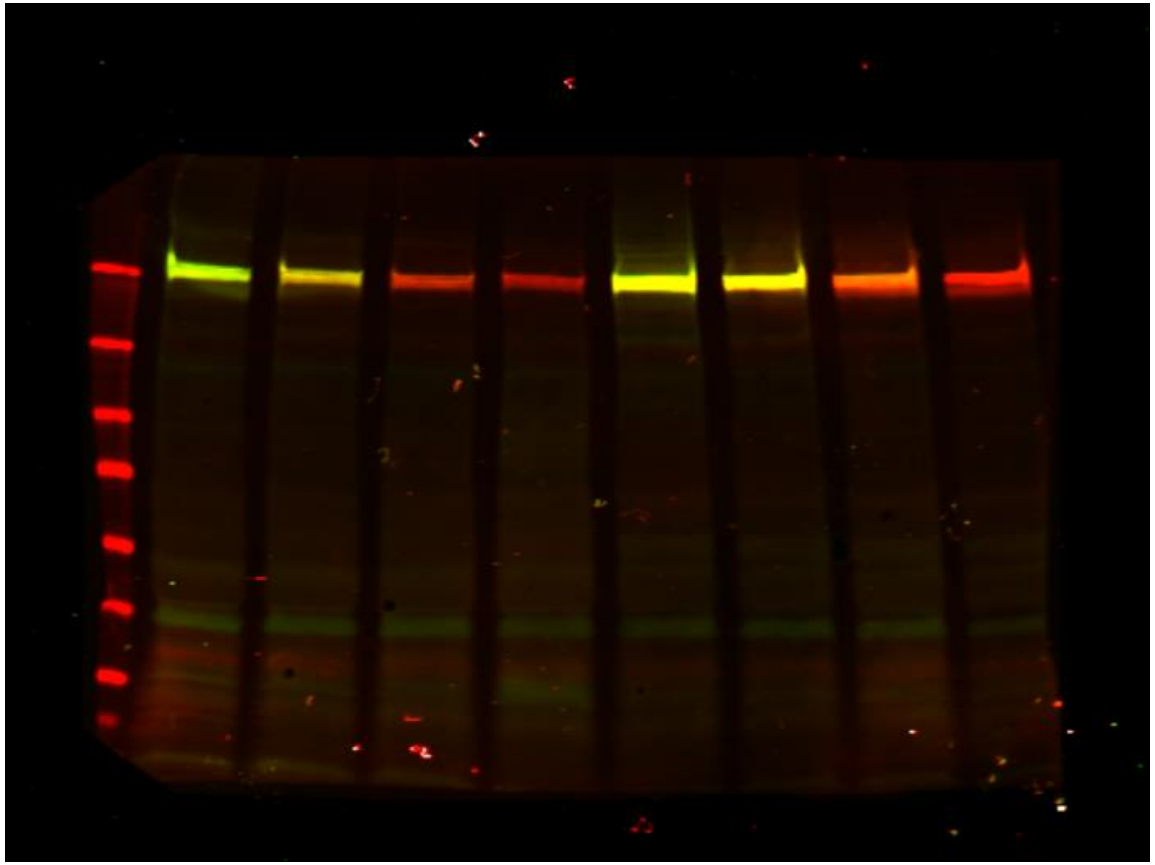

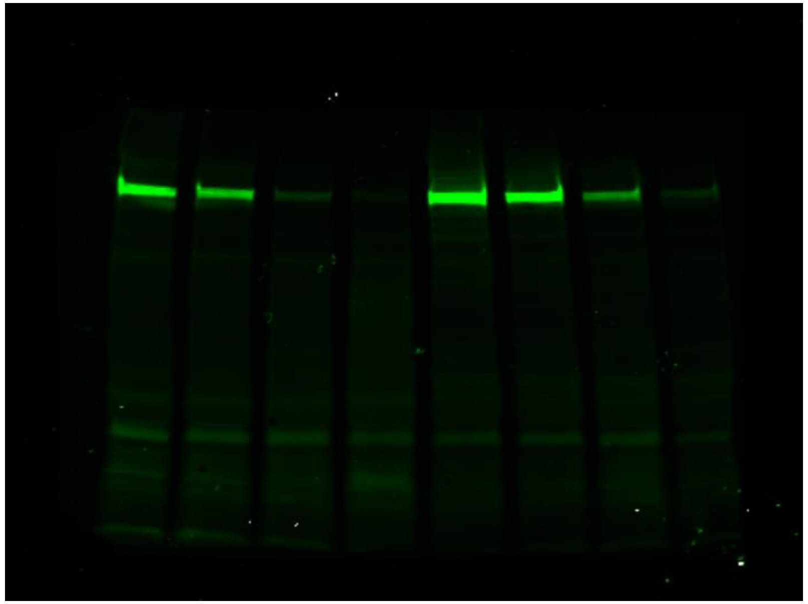

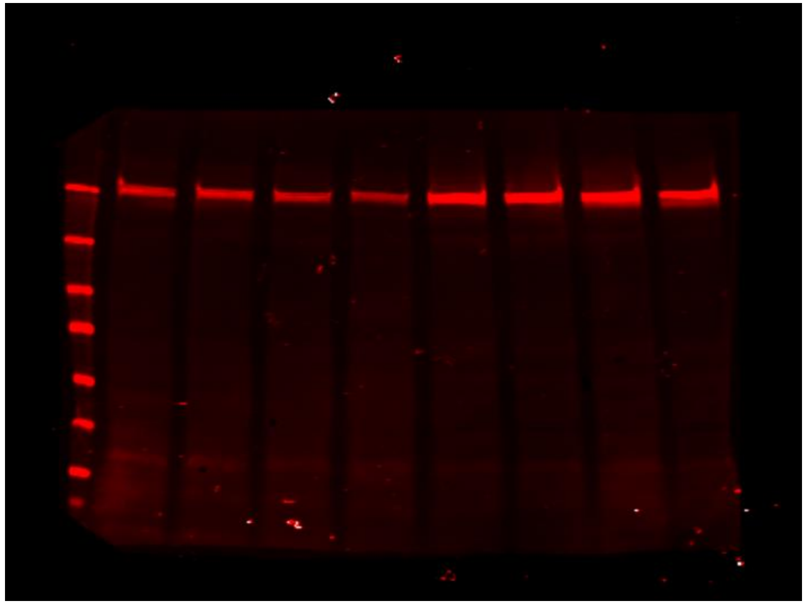

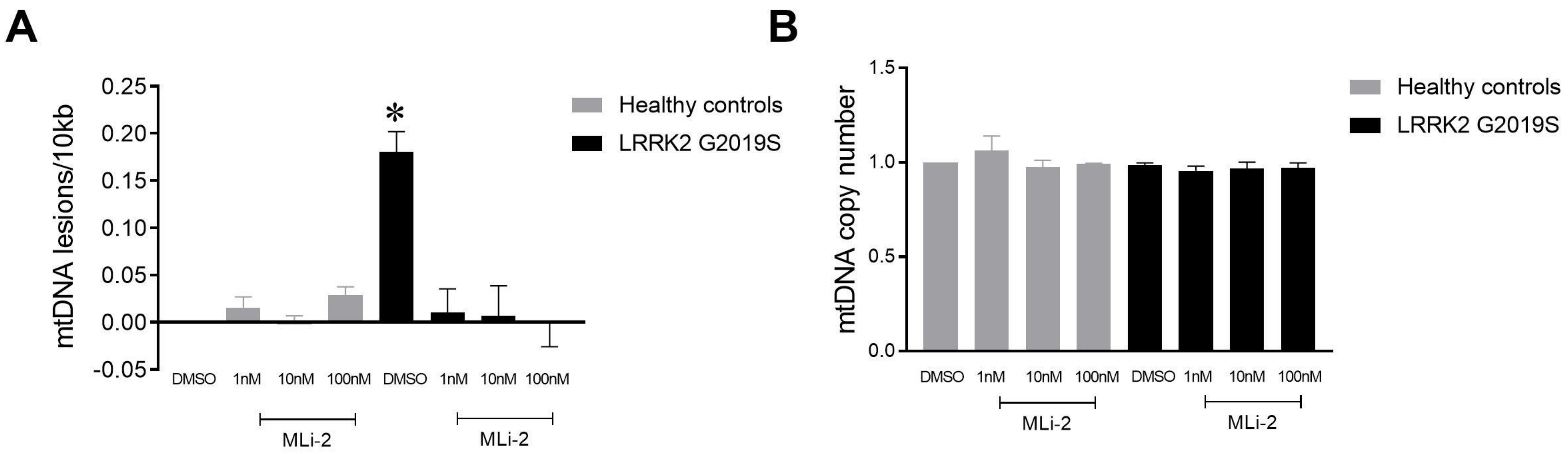

Supplemental Figure 16. Acute exposure to LRRK2 kinase inhibitors also restored mtDNA damage to basal levels. Healthy control or LRRK2 G2019S patient derived LCLs were treated with MLi-2 with doses ranging from 1-100nM for 1.5h. (A) Treatment with MLi-2 reversed mtDNA damage in LRRK2 G2019S-patient derived LCLs, with (B) no effect on mtDNA copy number. The PCR-based assay was performed in technical triplicate for each biological replicate. (**p* <0.001, determined by one-way ANOVA with a Tukey's post-hoc comparison). n = 3 biological replicates (6 cell lines total), each performed in technical replicate. Data are presented as mean ± SEM.
